# Review of the North American fauna of *Drymeia* Meigen (Diptera, Muscidae) and evaluation of DNA barcodes for species-level identification in the genus

**DOI:** 10.3897/zookeys.1024.60393

**Published:** 2021-03-15

**Authors:** Jade Savage, Vera S. Sorokina

**Affiliations:** 1 Bishop’s University, Sherbrooke, Quebec, Canada Bishop’s University Sherbrooke Canada; 2 Institute of Systematics and Ecology of Animals, Siberian Branch of the Russian Academy of Sciences, Novosibirsk, 630091, Russia Institute of Systematics and Ecology of Animals, Siberian Branch of the Russian Academy of Sciences Novosibirsk Russia

**Keywords:** Azeliinae, Barcoding Index Number (BIN), checklist, identification key, North America

## Abstract

The North American fauna of *Drymeia* Meigen was studied. Four new species are described (*Drymeia hucketti***sp. nov.**, *Drymeia ponti***sp. nov.**, *Drymeia vockerothi***sp. nov.**, *Drymeia woodorum***sp. nov.**), and three new synonymies are proposed: *Drymeia amnicola* (Huckett, 1966) (= *Drymeia rivalis* (Huckett, 1966), **syn. nov.**); *Drymeia glacialis* (Rondani, 1866) (= *Drymeia alpicola* (Rondani, 1871), **syn. nov.**); and *Drymeia spinitarsis* (Aldrich, 1918) (= *Drymeia longiseta* Sorokina & Pont, 2015, **syn. nov.**). An annotated checklist, DNA barcodes (when available), and keys for each sex of the 24 named species of North American *Drymeia* are provided. The utility of DNA barcodes for the identification of *Drymeia* species across a wide geographical range was explored using sequences from five countries. A match between morphology and DNA barcodes was found for 71% (22/31) of species studied (including three unnamed taxa). The remaining nine species clustered into two groups of taxa with very little interspecific variation within clusters (groups of two and seven species). Caution is advised against using DNA barcoding as the only determination tool for *Drymeia* material without prior knowledge of its limitations for certain species groups.

## Introduction

The genus *Drymeia* Meigen, 1826 includes more than 130 species of mid-sized muscids that can usually be distinguished from other members of the Muscidae by the combination of a dilated and densely setose gena, proclinate orbital bristles in the female, and three or more posterodorsal bristles on the hind tibia. An apical ventral spur of variable size can be found on the hind tibia of males in many species (see Fig. 2) and some taxa such as *Drymeia pribilofensis* (Malloch, 1919) and *D. spinitarsis* (Aldrich, 1918) exhibit elongated mouthparts (see Fig. 8A, B).

*Drymeia* is entirely restricted to the northern hemisphere and most species have an alpine and/or northern distribution. *Drymeia segnis* (Holmgren, 1883), for example, is among the few insect species found as far north as Qaanaaq (formerly known as Thule), Greenland (Michelsen 2015) and Eureka, Nunavut (Huckett 1965a). Eighty-eight species are recorded from the Palaearctic region (Hennig 1962a, b; Pont 1986; Fan 2008; Sorokina and Pont 2010, 2015; Michelsen 2011) and 34 from the Oriental region (Pont 1981; Shinonaga and Singh 1994; Shinonaga 2007; Xue et al. 2007, 2008, 2009; Fan 2008). With at least 75 recorded species, China has the highest number of *Drymeia* species of any country, and these are especially diverse in the Qinghai-Xizang (Tibetan) Plateau (56 species) (Xue et al. 2007, 2008, 2009; Fan 2008; Sorokina and Pont 2015). Prior to the present work, 21 species were recorded from North America, all found in the Nearctic region (Aldrich 1918; Malloch 1918; Huckett 1965a, b, 1966) with *Drymeia aterimma* (Wulp, 1896) being the only species also recorded from the Neotropical region (Mexico) (Nihei and Carvalho 2004; Carvalho et al. 2005).

While these flies can be collected using various passive collecting devices, net sweeping will often yield a good range of species since many can be found basking on sunny rocks or visiting flowers. Many species of *Drymeia* are known to consume pollen in addition to nectar, and Michelsen (2011) demonstrated that the morphology of the labellum has been modified to that effect in several species, including some found in the Nearctic region such as *Drymeia groenlandica* (Lundbeck, 1901) and *D. segnis*. The contribution of various *Drymeia* species to pollination services has not been widely investigated but in a study of the role of insect visitors in the pollination of *Dryas* in Northern Greenland, Tiusanen et al. (2016) demonstrated that in spite of comparable abundances and frequencies of flower visitation, *Spilogona sanctipauli* (Malloch, 1921) had a much stronger association with seed set than *D. segnis*.

Not much is known of the immature stages of *Drymeia*. Some larvae can be found in humus soil or cow dung and Skidmore (1985) reported that the larvae of the few species examined are dimorphic obligate carnivores with an extremely elongate and highly sclerotised cephaloskeleton.

The vast majority of North American species were originally described under *Pogonomyia* Rondani and, to a lesser extent, *Bebryx* Gistl, *Trichopticoides* Ringdahl, *Eupogonomyia* Malloch, and *Pogonomyioides* Malloch before these taxa were synonymised with *Drymeia* by Pont (1986), and Huckett and Vockeroth (1987). Malloch (1915, 1918, 1919, 1921) and Huckett (1965a, 1966) were the most important contributors to the Nearctic fauna of *Drymeia*, describing several species, 14 of which are still valid today (seven from each author).

Until recently, only *Drymeia alpicola* (Rondani, 1871) and *D. segnis* were recorded as having holarctic distributions. The works of Sorokina (2012) and Sorokina and Pont (2015) have uncovered several more: *Drymeia chillcotti* (Huckett, 1965), *D. firthiana* (Huckett, 1965), *D. groenlandica*, *D. quadrisetosa* (Malloch, 1919), *D. neoborealis* (Snyder, 1949), *D. pribilofensis*, and *D. setibasis* (Huckett, 1965). The northern distribution of many *Drymeia* species is conducive to a broader distribution range than seen in most southern taxa and we suspect that several additional species currently known only from the Nearctic region will eventually be found in the Palaearctic.

All taxonomic contributions to the North American fauna of *Drymeia* to date have focused on local fauna, making it difficult to identify specimens outside of these areas. Consequently, the first objective of this work was to develop comprehensive and well-illustrated keys to both sexes of all North American species of *Drymeia*. We also aimed to produce an annotated checklist of these species (including new synonymies and updated distribution ranges) and describe four new species. Finally, we explored the utility of DNA barcodes and Barcoding Index Number (BIN) assignments (Ratnasingham and Hebert 2013) for the identification of *Drymeia* species.

## Materials and methods

*Drymeia* specimens examined in this study are housed in the Academy of Natural Sciences of Philadelphia, Philadelphia, USA (**ANSP**), the Bishop’s University Insect Collection, Sherbrooke, Canada (**BUIC**), the California Academy of Sciences, San Francisco, USA (**CAS**), the Canadian National Collection of Insects, Arachnids and Nematodes, Ottawa, Canada (**CNC**), the Centre for Biodiversity Genomics, Guelph, Canada (**BIOUG**), the Essig Museum of Entomology, University of California, Berkeley, USA (**EMEC**), the Finnish Museum of Natural History, Helsinki, Finland (**MZH**), the Illinois Natural History Survey Insect Collection, Champaign, USA (**INHS**); the Lyman Entomological Museum, McGill University, Ste-Anne-de-Bellevue, Canada (**LEM**), the R.M. Bohart Museum of Entomology, University of California, Davis, USA (**UCDC**), the National Museum of Natural History, Washington D.C., USA (**USNM**), and the Siberian Zoological Museum of the Institute of Systematics and Ecology of Animals, Novosibirsk, Russia (**SZMN**). Limits of the Nearctic and Neotropical regions follow Morrone (2014).

### Morphology

Morphological terms follow McAlpine (1981) for external structures and Savage and Wheeler (2004) for male genitalia. Body length was measured in millimetres (mm) from the anterior margin of the head without antenna to the apex of the abdomen. Frons length was measured between lower margin of frons and upper margin of ocelli triangle. Genitalia were cleared using lactic acid and placed in a microvial pinned under the specimen following dissection. Whole specimens and external structures were photographed with a Leica DFC450 or Leica DFC5400 camera using Leica Application suite X (LAX) and images were stacked using LAS X and Zerene Stacker 1.04. Male genitalia were photographed using a Luminera 1 camera and images were stacked using Helicon Focus 6.3.0 (Helicon Soft Ltd., Kharkov, Ukraine). Final image editing and plate assembly was completed in Adobe Photoshop CS.

The following abbreviations are used in the text:

**a** anterior;

**acr** acrostichal;

**ad** anterodorsal;

**av** anteroventral;

**dc** dorsocentral;

**p** posterior;

**pd** posterodorsal;

**pv** posteroventral;

**v** ventral;

**F** femur;

**T** tibia.

Label data of primary type material examined are presented verbatim, with “/” indicating a change of line and “;” a change in label.

### DNA barcoding

The Barcode Index Number (BIN) system uses the Refined Single Linkage (RESL) algorithm to group COI sequences in the Barcode of Life Data System (BOLD) (http://boldsystems.org) into genetic clusters (BINs) representing proxies for species (Ratnasingham and Hebert 2013). While the present work deals mainly with the North American fauna, we assessed the match between BIN assignment and the morphology of current specie concepts globally and therefore included all available sequences regardless of geographical origins. This broad geographical approach had the potential to uncover new species (and/or synonymies) and yield new data on species distribution ranges. If the morphology of specimens from BINs that could not be assigned to a named species was clearly unique and the material in good enough condition, the species was described.

All DNA barcodes (Folmer region of COI gene) were sequenced at the University of Guelph Biodiversity Institute of Ontario following protocols published in Hajibabaei et al. (2005) and the LepF1/LepR1 (Hebert et al. 2004) and LCO1490_t1/HCO2198_t1 (Foottit et al. 2009) primers were used for amplification. All sequences were at least 550 base pair long with zero ambiguous base and these were aligned in BOLD) using the BOLD Aligner tool (Amino Acid based HHM). A neighbour-joining tree was built in BOLD to provide a graphical representation of pairwise distance between specimens in the data set, detect potential anomalies, and examine BIN assignments. Uncorrected pairwise distances (p-distance) were calculated in MEGA X (Kumar et al. 2018).

In the public dataset "Drymeia specimens examined for Savage and Sorokina 2021" (dx.doi.org/10.5883/DS-DRYNEW) available on BOLD. Specimen details, including BIN assignment, sex, and GenBank accession numbers for this data set can also be found in Suppl. material 1: Table S1. All specimens were examined by the authors except for the only representative of *Drymeia hamata* (Fallén, 1823) (BOLD process ID: GBDP5828-09); we are, however, highly confident in the identity of that specimen since the unique elongated mouthparts characteristic of the species are clearly visible in the image of the male for this record.

## Results

### Drymeia

Taxon classificationAnimaliaDipteraMuscidae

Meigen, 1826

B04F3E18-1E33-5F83-86A3-0BF91E1F233C

Drymeia Meigen, 1826: 204. Type species: Drymeia obscura Meigen, 1826 [= Musca hamata Fallén, 1823] (monotypy).
Drymia Agassiz, 1847: 130. Unjustified emendation of Drymeia Meigen.
Eriphia Meigen, 1826: 206. [Junior homonym of Eriphia Latreille, 1817: Crustacea] Type species: Eriphia cinerea Meigen, 1826 (monotypy).
Bebryx Gistl, 1848: ix. [Replacement name for Eriphia Meigen, 1826] Type species: Eriphia cinerea Meigen, 1826 (automatic).
Pogonomyia Rondani, 1871 [1870]: 336. Type species: Pogonomyia alpicola Rondani, 1871 (monotypy).
Neoeriphia Schnabl & Dziedzicki, 1911: 195 [as subgenus of Eriphia Meigen, 1826]. Type species: Eriphia metatarsata Stein, 1907 (monotypy).
Neopogonomyia Schnabl & Dziedzicki, 1911: 198 [as subgenus of Pogonomyia Rondani, 1871]. Type species: Aspilia brumalis Rondani, 1866, by designation of Séguy, 1923, Faune Fr., 6: 295. [A. brumalis is listed by Schnabl and Dziedzicki as a junior synonym of *meadei*, and *meadei* is the first of two included species.]
Pogonomyioides Malloch, 1919: 67. Type species: Pogonomyioides atrata Malloch, 1919 [= Aricia segnis Holmgren, 1883] (original designation).
Eupogonomyia Malloch, 1921: 178. Type species: Eupogonomyia pribilofensis Malloch, 1921 (original designation).
Trichopticoides Ringdahl, 1931: 173. Type species: Musca decolor Fallén, 1824 [= Musca vicanus Harris, 1780] (monotypy).

#### Remarks.

While the exact systematic position of *Drymeia* remains debated (Savage and Wheeler 2004; Kutty et al. 2014) its placement within the tribe Azeliini, subfamily Azeliinae is broadly accepted (Carvalho et al. 2005; Sorokina and Pont 2010, 2015; Gregor et al. 2016). Monotypic until 1986, the limits of the genus were greatly expanded by a series of synonymies by Pont (1986) and Huckett and Vockeroth (1987) (see Sorokina and Pont 2015 for details). The monophyly of *Drymeia* is well-supported (Savage and Wheeler 2004; Michelsen 2011) and a detailed generic description is provided in Savage and Wheeler (2004). Members of the group can be distinguished from other azeliines by the combination of at least three posterodorsal setae on the hind tibia (these not restricted to the apical 1/2), hind coxa bare on the posterodorsal surface, a black haltere, and gena usually broad with numerous upcurved setae. Females have the cruciate frontal setae present in all Nearctic species and the proclinate lower orbital setae often slightly lateroclinate. Males of most species in the Nearctic region exhibit a small to well-developed apical ventral projection on the hind tibia (Fig. 2A, C, F, G, H, I) and a wide range of striking leg armature, especially on the mid femur (Fig. 6).

### Key to males of North American species of *Drymeia* Meigen

(males of *D. latifrons* (Malloch) unknown)

**Table:** 

1	Calypter black. Recorded only from Mexico	**2**
–	Calypter pale brown to yellow. Not recorded from Mexico	**3**
2	Frons at narrowest point at least 1.5 × as wide as ocellar triangle and with frontal vitta widely exposed (Fig. 1A); T3 with ventral apical process short but clearly visible and with apical *pv* absent (Fig. 2A); T2 usually with 2 *ad*	***ponti* sp. nov.**
–	Frons narrower than ocellar triangle and with fronto-orbital plates usually touching at narrowest point (Fig. 1B); ventral apex of T3 transverse, without apical process and with apical *pv* present (Fig. 2B); T2 usually with 1 *ad*	***aterrima* (Wulp)**
3	Anepimeron haired	**4**
–	Anepimeron bare	**5**
4	Epandrium clotted with very long curly hair forming a distinctive brush at tip of abdomen (Fig. 3A); fore coxa normal, not prolonged apically into a knob-like structure; F2 with a row of long straight *av* of decreasing length on apical 1/3	***chillcotti* (Huckett)**
–	Epandrium with regular setae; fore coxa prolonged into a knob-like structure (Fig. 3B); F2 with a ventral clump of long curly hair positioned ca. 1/3 from apex (Fig. 3B)	***segnis* (Holmgren)**
5	Prealar reduced or absent; if visible, then no longer than 1/2 the length of posterior notopleural bristle; 2+4 *dc*	**6**
–	Prealar strong, approximately as long as posterior notopleural bristle; *dc* variable	**12**
6	Lower margin of face strongly projecting beyond lower level of frons (Fig. 4A); F2 with a line of long dense *av* starting prebasad and ending before apical 1/4; outer claw of fore tarsus enlarged (Fig. 5A)	***pribilofensis* (Malloch)**
–	Lower margin of face not or only weakly projecting beyond lower level of frons; F2 not as above; outer claw of fore tarsus not strongly enlarged (sometimes slightly larger in *D. setibasis*)	**7**
7	T3 with ventral apical process very strongly developed, longer than width of hind tibia (Fig. 2C); F2 with tuft of long *av* near apex	***groenlandica* (Lundbeck)**
–	T3 with apical process short to well-developed but always much shorter than maximum width of hind tibia (Fig. 2D, F, J); F2 without tuft of long *av* near apex	**8**
8	F2 with 1 or 2 short stout ventral spines near middle (Fig. 6A)	***cantabrigensis* (Huckett)**
–	F2 without short stout spines near middle but sometimes with short ventral spines closer to base	**9**
9	F2 with a very strong clump of curved *av* near the very base (Fig. 6B); small species (4.8–6.2 mm)	***setibasis* (Huckett)**
–	F2 at most with a few long delicate *av* on basal 1/3 (Fig. 6C); size variable (7.7–9.5 mm)	**10**
10	Fore coxa with a patch of 4 or 5 very long, strong apical bristles, at least as long as length of coxa; smaller species (5.5 mm)	***woodorum* sp. nov.**
–	Fore coxa without long bristles, only with short spines; larger species (7.7–9.5 mm)	**11**
11	Frons broad with black frontal vitta exposed and at least 2 × as wide as fore ocellus (Fig. 1C); parafacial broad in lateral view (Fig. 4B); *acr* weak, no stronger than ground setae	***neoborealis* (Snyder)**
–	Frons narrow with fronto-orbital plates touching at narrowest part (Fig. 1D); parafacial narrow in lateral view (Fig. 4C); *acr* strong, distinct from ground setae	***vockerothi* sp. nov.**
12	Parafacial broad in lateral view, wider than length of first flagellomere midpoint (Fig. 4D, E); apex of fore tarsomere 1 ending in a sharp ventral spike (less developed in *D. spinitarsis*) (Fig. 5D); *dc* variable	**13**
–	Parafacial in lateral view no wider than width of first flagellomere midpoint (Fig. 4F–H); ventral apex of tarsomere 1 transverse, not ending in a spike; 2+3 *dc*	**15**
13	Frons broad with black frontal vitta exposed and at least as wide as fore ocellus at narrowest point; fore and mid coxa with very long fine hair, crinkled near apex; mid tarsomere 1 with spines in *av* and *pv* rows long, some longer than width of tarsomere (Fig. 5B); T3 with ventral apical process short but clearly visible	***spinitarsis* (Aldrich)**
–	Frons narrow with fronto-orbital plates touching at narrowest point; hairs of fore and mid coxa straight, not as above; mid tarsomere 1 with spines in *av* and *pv* rows shorter than width of tarsomere; T3 with ventral apical process present or absent	**14**
14	F2 with complete row of *av* and with 2 or 3 long and strong *v* about 1/3 from apex (Fig. 6F); T2 without *av* and *ad*; T3 with ventral apical process short but clearly visible (Fig. 2G); 2+4 *dc*	***quadrisetosa* (Malloch)**
–	F2 with complete row of *av* but without long and strong *v* about 1/3 from apex (Fig. 6E); T2 with 1–4 *av* (these sometimes weak) and 1–5 *ad*; T3 with ventral apical process reduced (Fig. 2H), sometimes absent; *dc* usually 2+4 but occasionally 2+3	***glacialis* (Rondani)**
15	Wing with membrane and veins conspicuously yellow over most of the surface; T3 with a complete row of numerous short, sub-equal *ad*, no longer than diameter of tibia	***flavinervis* (Malloch)**
–	Wing usually hyaline or infuscated brown but if yellowish, then row of *ad* on T3 not as above	**16**
16	F2 with *av* and *pv* rows short and irregular, restricted to apical 1/2 and with bristles no longer than diameter of femur (Fig. 6I); T3 with ventral apical process absent or very much reduced (Fig. 2E)	***similis* (Malloch)**
–	F2 with *av* and/or *pv* rows variable, but always with some (or many) bristles longer than basal diameter of femur (as in Fig. 6K, L); T3 ventral apical process short but usually distinct (Fig. 2I)	**17**
17	F2 with both *av* (Fig. 6D) and *pv* rows complete and erect	**18**
–	F2 with either *av* row incomplete (as in Fig. 6J) or with bristles curved on basal 1/2 (Fig. 6K), *pv* row variable.	**19**
18	Prementum dusted; scutum dusted, abdomen grey dusted except for most of tergite I+II, a wide central patch on tergite III and central vittae on tergites IV and V (Fig. 7A); F2 and/or F3 without long fine and slightly crinkled *p-pv* on basal 1/4.	***firthiana* (Huckett)**
–	Prementum undusted, shiny; scutum and abdomen mostly shiny; F2 and/or F3 usually with a few long fine and slightly crinkled *p-pv* on basal 1/4 (Fig. 6D)	***profrontalis* (Huckett)**
19	F2 with *pv* row incomplete, with 4–7 very long *pv* on apical 1/2, these at least 2 × as long as width of femur	**20**
–	F2 with *pv* row complete, with some bristles longer than width of femur at least on basal 1/2 (Fig. 6G, H, L)	**21**
20	Lower margin of face projecting slightly beyond lower level of frons (Fig. 16A); arista with hairs restricted mostly to basal 1/2 and with no or few dorsal setae on apical 1/2; abdomen lightly dusted and mostly shiny, tergites without distinct dark central vittae (Fig. 7B)	***hucketti* sp. nov.**
–	Lower margin of face not projecting beyond lower level of frons (Fig. 4H); arista with hairs distributed along full length; abdomen grey/brown dusted with clear dark central vittae on tergites III–V (Fig. 7C)	***minor* (Malloch)**
21	F2 with *pv* row sparse and delicate, with only a few bristles longer than width of femur (Fig. 6G); scutum and abdomen mostly dusted	***santamonicae* (Huckett)**
–	F2 with *pv* row regular and thicker than above, with many bristles longer than width of femur on basal 2/3 (Fig. 6H, L); scutum and abdomen mostly shiny	**22**
22	T2 without *ad* on apical 1/3, California records doubtful (see remarks under *D. aldrichi*)	***aldrichi* (Malloch)**
–	T2 usually with 1 or 2 *ad* present on apical 1/3 (these sometimes absent), recorded only from California	***amnicola* (Huckett)**

### Key to females of North American species of *Drymeia* Meigen

**Table:** 

1	Prealar bristle present and strong, as long as 2^nd^ notopleural bristle	**2**
–	Prealar bristle absent or short, always much shorter than 2^nd^ notopleural bristle	**19**
2	Parafacial in lateral view broader than width of first flagellomere (Fig. 8A); F2 with a complete row of long strong *av* and a complete row of long, delicate, irregular *pv*; 2+3–4 *dc.* Larger species, 6.2–9.5 mm long.	**3**
–	Parafacial in lateral view variable; F2 not as above; 2+3 *dc.* Size variable but usually < 7.5 mm	**5**
3	Mid tarsomere 1 with spines in *av* and *pv* rows long, some longer than width of tarsomere (Fig. 5C); head in lateral view higher than wide (Fig. 8A)	***spinitarsis* (Aldrich)**
–	Mid tarsomere 1 with spines in *av* and *pv* rows shorter than 1/2 the width of tarsomere; head in lateral view no higher than wide (as in Fig. 4E)	**4**
4	Veins of basal region of wing except costa yellowish to pale brown	***quadrisetosa* (Malloch)**
–	Veins of basal region of wing dark brown to yellowish	***glacialis* (Rondani)** [Nearctic females of *D. glacialis* generally exhibit a darker wing base than those of *D. quadrisetosa* but females of these two species are difficult to distinguish (see Remarks under *D. glacialis* for additional details)]
5	Entire wing membrane and all veins rich yellow (costa brownish only near wing tip) (Fig. 9A); parafacial in anterior view with a small to medium-size undusted shiny patch near antennal base	***flavinervis* (Malloch)**
–	Wing membrane never rich yellow throughout most of the surface and most veins brownish near apex; parafacial shining or dusted	**6**
6	Prementum dusted and no longer than palpus; body light grey dusted; T2 with 2 or 3 *ad*; T3 with apical *pv* short or absent; parafacial and profrons in anterior view entirely dusted.	***firthiana* (Huckett)**
–	Prementum shiny, usually longer than palpus; body mostly undusted and shiny except in *D. santamonicae*; T2 with *ad* variable; T3 with apical *pv* present or absent; dusting of parafacial and profrons variable	**7**
7	T2 with at least 1 *av*, costal spinules usually long and strong with costal spine at least 1.5 × as long as surrounding spinules (Fig. 15A)	**8**
–	T2 usually without *av*; costal spinules and costal spine variable	**10**
8	T3 with apical *pv* usually absent but if visible, then no longer than 1/2 the length of apical *av*; arista with longest hair no longer than base or arista; fore tarsomere 5 distinctly flattened (Fig. 10A). Recorded only from Mexico	***ponti* sp. nov. (part, usually without *av* on T2)**
–	T3 with apical *pv* present, at least 1/2 as long as apical *av*; arista with longest hair usually slightly longer than base of arista; fore tarsomere 5 at most slightly flattened (Fig. 10C)	**9**
9	Parafacial entirely dusted or with small undusted line near base of antenna (Fig. 11A); frons narrow, approximately 1.4 × as long as wide (Fig. 11A); wing membrane with pale yellow tinge	***similis* Malloch (part, usually without *av* on T2)**
–	Parafacial with large undusted shiny patch near base of antenna (Fig. 11B); frons wide, approximately 0.9 × as long as wide (Fig. 11B); wing membrane with pale brown tinge	***latifrons* (Malloch)**
10	Parafacial entirely dusted or with small undusted line near base of antenna (Fig. 11A); ocellar triangle mostly undusted and shiny; costal spine strong, at least 2 × as long as other costal setae; F2 without strong long *av* on apical 1/2, only with weak setae; wing yellow near base	***similis* (Malloch) (part)**
–	With different combination of characters	**11**
11	Fronto-orbital plates and ocellar triangle mostly undusted and shiny (Fig. 11C); parafacial with large undusted shiny patch near base of antenna expanded ventrally (Fig. 11C); fore tarsomere 5 moderately to distinctly flattened (Fig. 10D); scutum and abdomen black and shiny; recorded only from California	***profrontalis* (Huckett)**
–	Fronto-orbital plates mostly to completely dusted (Fig. 11A, D, E); other features variable	**12**
12	Parafacial in anterior view with large undusted shiny patch near base of antenna reaching up to or almost up to eye (Fig. 11E)	**13**
–	Parafacial in anterior view completely or mostly dusted, at most with a small shiny patch near base of antenna (Fig. 11D)	**15**
13	Scutum and abdomen dark grey with thin brownish grey pollen; ocellar triangle entirely dusted; parafacial in lateral view no broader than width of first flagellomere at midpoint; recorded only from California	***santamonicae* (Huckett)**
–	Scutum and abdomen black and mostly shiny (Fig. 18A); ocellar triangle partially to completely shiny; parafacial width and distribution variable	**14**
14	Parafacial in lateral view no broader than width of first flagellomere (as in Fig. 15A); wing veins and membrane mostly brown; F2 variable but usually with 1–3 *av*, recorded only from California	***amnicola* (Huckett) (part)**
–	Parafacial in lateral view broader than width of first flagellomere (Fig. 18A); Wing veins yellow at least near base, membrane deep yellow near base, the rest pale yellow to pale brown (Fig. 18A); F2 variable but usually with 4 or 5 *av*; not recorded from California	***hucketti* sp. nov.**
15	Fore tarsomere 5 moderately to distinctively flattened (Fig. 10A, B)	**16**
–	Fore tarsomere 5 not or only slightly flattened (Fig. 10E)	**17**
16	Costal spinules long and strong with costal spine 2 × as long as surrounding spinules (Fig. 15A); ocellar triangle completely dusted (Fig. 11D); lower margin of face projecting very slightly beyond lower level of frons (Fig. 15B); T3 with apical *pv* usually absent but if visible, then no longer than 1/2 the length of apical *av*; recorded only from Mexico	***ponti* sp. nov. (part)**
–	Costal spinules not strongly developed, with costal spine only slightly larger than surrounding spinules (Fig. 9B); ocellar triangle only partially dusted, with some shiny areas; lower margin of face not projecting beyond lower level of frons; T3 with long strong apical *pv* as long or slightly shorter than apical *av*; not recorded from Mexico	***aldrichi* (Malloch)**
17	Arista with longest hair 1.5–2.0 × width of base of arista (Fig. 12B); F2 usually with 1 (sometimes 2) distinct *av* on apical 1/3, as long or slightly longer than width of femur; recorded from Mexico and USA	***aterrima* (Wulp)**
–	Arista with longest hair as long as base of arista (Fig. 12C); F2 *av* variable; not recorded from Mexico	**18**
18	Larger species, 6.2–7.3 mm; F2 usually with av reduced on apical 1/2, these no longer than width of femur; recorded only from California	***amnicola* (Huckett) (part)**
–	Smaller species, 4.5–5.7 mm; F2 usually with 2 strong *av* on apical 1/2, longer than width of femur; broader distribution	***minor* (Malloch)**
19	Anepimeron and/or katepimeron with hairs	**20**
–	Anepimeron and katepimeron both bare	**21**
20	F2 with 1 or 2 strong *av* setae on apical 1/2, as strong as or stronger than *av* near base; lower margin of face not projecting beyond lower level of frons; usually a larger species 7.4–9.3 mm.	***chillcotti* (Huckett)**
–	F2 without or with only a short weak *av* in apical 1/2; lower margin of face slightly projecting beyond lower level of frons; usually a smaller species 6.0–8.5 mm	***segnis* (Holmgren)**
21	Parafacial in lateral view narrow at midpoint, < 1/2 the width of first flagellomere (Figs 21A, 24A); proboscis short and wide (as in Fig. 19A); presutural acrostical bristles distinct from ground setulae (Figs 21B, 24B)	**22**
–	Parafacial in lateral view wide at midpoint, at least as wide as first flagellomere (Fig. 8B, C); proboscis slender and tubular (Fig. 8B); presutural acrostical bristles weak	**23**
22	Large species (7.5–9.0 mm) (Fig. 21A); scutum and thorax subshiny black, without marks (Fig. 21A); setulae present near base of posterior notopleural bristle	***vockerothi* sp. nov.**
–	Small species (4.9–5.5 mm) (Fig. 24A); scutum and thorax covered with thick grey dust (Fig. 24B, C); setulae absent near base of posterior notopleural bristle	***woodorum* sp. nov.**
23	T2 with *av* present	***setibasis* (Huckett) (part)**
–	T2 with *av* absent	**24**
24	Lower margin of face not projecting beyond lower level of frons; prealar stronger than ground setulae but always much weaker and shorter than posterior notopleural bristle	**25**
–	Lower margin of face projecting beyond lower level of frons; prealar usually absent (a weak prealar sometimes visible in *D. cantabrigensis*)	**26**
25	F2 with longest *av* on apical 1/3 as long as width of femur; abdomen lightly dusted, subshiny	***groenlandica* (Lundbeck)**
–	F2 with longest *av* on apical 1/3 shorter than width of femur; abdomen heavily dusted	***neoborealis* (Snyder)**
26	Lower margin of face usually strongly projecting beyond lower level of frons (Fig. 8B); F2 with *av* and *pv* rows usually complete or near complete; T2 with all *pd* shorter than preapical *d*; proboscis with prementum longer than palpus (Fig. 8B); larger species, at least 6.5 mm long	***pribilofensis* (Malloch)**
–	Lower margin of face projecting only slightly beyond lower level of frons (Fig. 8C); F2 with *av* and *pv* rows usually restricted to basal 1/2; T2 with most *pd* as long or longer than preapical *d*; proboscis with prementum no longer than palpus, usually as long as palpus; smaller species, < 6.2 mm long	***setibasis* (Huckett) (part), *cantabrigensis* (Huckett)** [Some females of *D. pribilofensis* occasionally do not exhibit the full combination of characters listed in the key and may therefore be difficult to separate from *D. setibasis* and *D. cantabrigensis*.]

**Figure 1.**
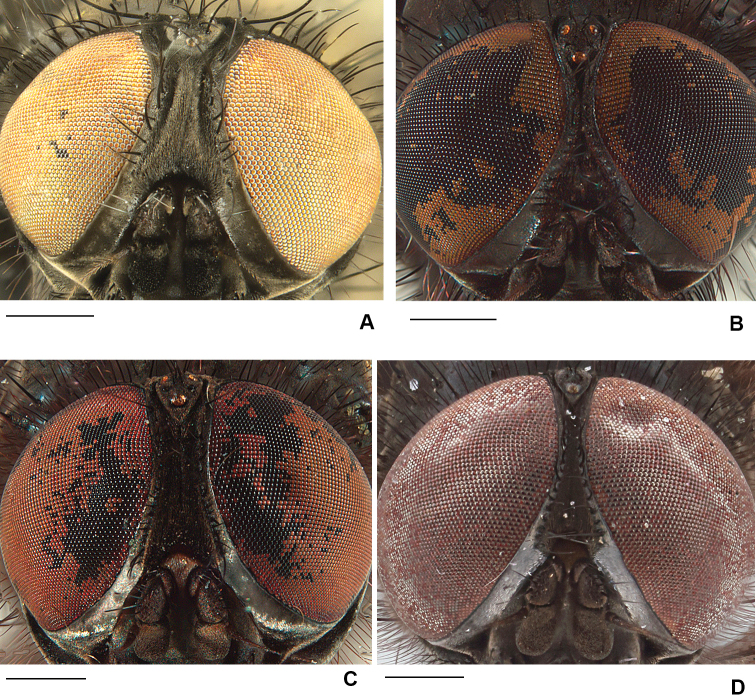
Male frons, frontal view **A***D. ponti***B***D. aterrima***C***D. neoborealis***D***D. vockerothi*. Scale bars: 0.5 mm.

### Annotated checklist and new species descriptions

#### Drymeia aldrichi

Taxon classificationAnimaliaDipteraMuscidae

(Malloch, 1918)

164028E0-95C4-5DF1-A726-EE61C4114AA3

Figs 6L , 9B , 10B

Pogonomyia aldrichi Malloch, 1918: 281.
Pogonomyia unicolor , Stein, 1920: 22.

##### Type material examined.

*Pogonomyia aldrichi* – ***Holotype*** male labelled “INHS/Insect Collection/238,899; “Moscow Ida/v.22.13”; “TYPE/ Pogonomyia/ aldrichi/♂ [red]” (INHS).

##### Other material examined.

2 females: Nearctic: **USA**: Idaho: Moscow (INHS).

##### Distribution.

Nearctic: Canada (Alberta), USA (Washington to California (see remarks), Wyoming).

##### DNA barcode.

None available.

##### Remarks.

While California was listed in the distribution of *D. aldrichi* by Huckett (1965b), the species was not included in a subsequent work by the same author on the fauna of California (Huckett 1975). We therefore suspect that early records of *D. aldrichi* from California belong instead to another species, possibly the very similar *D. amnicola*, described by Huckett in 1966. As mentioned in the DNA barcoding section below, both the distribution and the limits of *D. aldrichi* are currently uncertain and additional data will be required to clarify the issue.

#### Drymeia amnicola

Taxon classificationAnimaliaDipteraMuscidae

(Huckett, 1966)

4F9560DD-39A3-533D-8B35-02A685F2D68D

**Figure 2.**
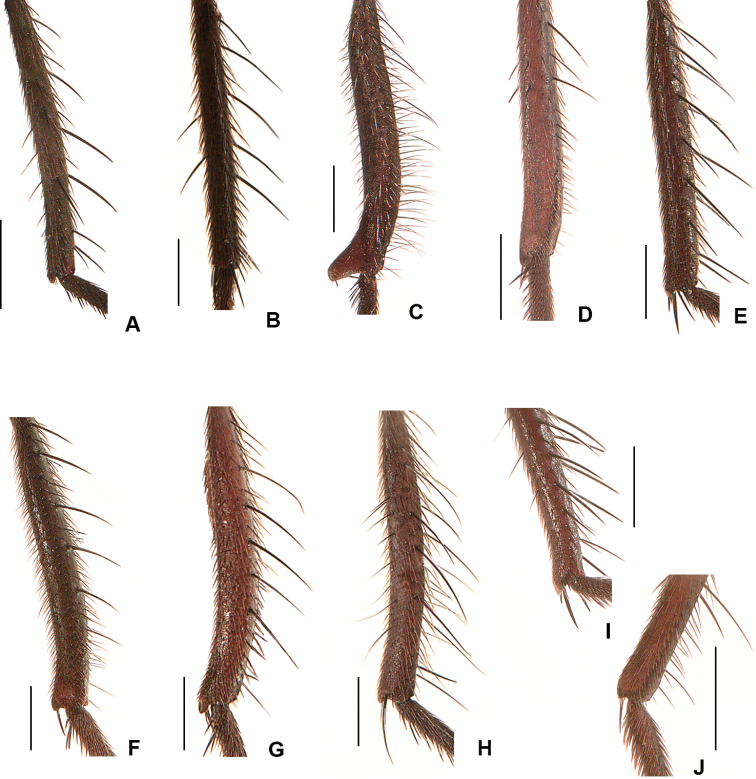
Male hind tibia, dorsal view **A***D. ponti***B***D. aterrima***C***D. groenlandica***D***D. setibasis***E***D. similis***F***D. vockerothi***G***D. quadrisetosa***H***D. glacialis***I***D. hucketti***J***D. woodorum*. Scale bars: 0.5 mm.

Figs 2B , 4G , 6H, J

Pogonomyia amnicola Huckett, 1966: 291.
Pogonomyia rivalis Huckett, 1966: 293. syn. nov.

##### Type material examined.

*Pogonomyia amnicola* – ***Holotype*** male labelled “Sardine Crk./Mono Co./ Elev. 8500/ Cal. VI-28-51”; “J.W. MacSwain/ Collector”; “Pogonomyia/ amnicola/ n. sp./ holotype [red]”; “California Academy/ of Sciences/ Type No. 10149” (CAS). ***Allotype*** female labelled “Sardine Crk./Mono Co./ Elev. 8500/ Cal. VI-28-51”; “J.W. MacSwain/ Collector”; “Pogonomyia/ amnicola/ n. sp./ allotype [orange]”; “U.C. Berkeley/EMEC 69,278” (EMEC).

***Paratypes*:** all with “Pogonomyia/ amnicola/ n. sp./ paratype [blue]”. 2 males, “Sardine Crk./Mono Co./ Elev. 8500/ Cal. VI-28-51”; “J.W. MacSwain/ Collector” (EMEC). 5 males, 1 female, same as previous except “CA Downing Collector”. 9 females, 3 males, same as previous except “A.T. McClay/ Collector” (UCDC).14 females, 7 males, same as previous except “S.M. Kappos/ Collector”. 1 male, same as previous except “R.W. Morgan/ Collector”. 1 female, same as previous except “D.P. Lawfer/ Collector”. 2 males, 1 female, same as previous except VII-11-51; “A.T. McClay/ Collector”. 4 females, same as previous except VII-12-51. 14 males and 11 females, same as previous except “Sardine Creek/ Mono Co Cal./ VI-28-1951. 1 female, “Hope Valley/ Alpine Co/ Calif. VII-9-48”; “S.A. Sher/ Collector” (EMEC); 1 female, same as previous except “D. Carter/ Collector”. 6 females, “4 miles north/ Silver Lake Cal./ Amador Co./ VII-25. 1955”; “E.I. Schlinger/ Collector” (UCDC). 1 male, same as previous except “J. C.Downey/ Collector”; 1 male, Echo Lake Cal./ Eldorado Co./ VII-23-1955”; “E.I. Schlinger/ Collector” (UCDC). 1 male, same as previous except “J. C.Downey/ Collector”. 1 female, “Winnemucca/ Lk. Alpine Co/ Cal. VI-30-1959”; “R.M. Bohart/ Collector” (UCDC). 1 male, same as previous except “P.M. Maran/ Collector. 2 females, “North Lake/ Inyo Co. Calif/ VI-30-61”; “J.S. Buckett/ Collector” (UCDC). 1 female, “5 miles east/ Weber Lake/ Cal. Sierra Co./ VII.29.1955”; “R.W. Bushing/ Collector” (UCDC). 1 female, same as previous except VII.30.1955; “E. A. Kurta/ Collector”. 1 male and 1 female, “1 mi.5/ Saddlebag L./ Mono. Co. Cal./ VII.15-1961”; “D. R. Miller/ Collector” (UCDC). 1 female, “Luther Pass Cal./ Grass Lake/ Eldorado Co./ VII-24-1955”; “J.C. Downey/ Collector” (UCDC). 1 female, “Wood Lk. Cal./ Alpine Co./ VII-16 1960”; “C.G. Moore/ Collector” (UCDC). 1 male, “Sonora Pass/ Cal Elev 9624/ VII.17 1953”; “R.M. Bohart/ Collector” (UCDC). 1 male, “Strawberry/ Tuolumne Co/ Calif. VII.15 51”; “A.T. McClay/ Collector” (UCDC).

*Pogonomyia rivalis* – ***Allotype*** female labelled “Sardine Crk./Mono Co./ Elev. 8500/ Cal. VI-28-51”; “S.M. Kappos/ Collector”; “Pogonomyia rivalis/ n. sp./ Allotype [orange]” (UCDC). ***Paratypes***: all with “Pogonomyia/ rivalis/ n. sp./ paratype [blue]”. 3 males and 1 female, same as allotype. 1 female, same as previous except “J.W. MacSwain/ Collector”. 2 females and 1 male, same as previous except (EMEC). 1 male, same as previous except “C.A. Downing/ Collector”. 1 female, same as previous except “D.P. Lawfer/ Collector” (UCDC). 2 males and 1 female, same as previous except “Sardine Creek/ Mono Co Cal./ VI.28.1951”; “A.T. McClay/ Collector”. 1 female, same as previous except VII-8-51.

##### Other material examined.

1 male, 7 females: Nearctic: **USA**: California: Leavitt Mdw, 1 mi. S saddlebag Lake, Sardine Creek (EMEC, UCDC).

##### Distribution.

Nearctic: USA (California).

##### DNA Barcode.

None available.

##### Remarks.

In his original descriptions, Huckett (1966) relied on subtle differences in variable characters to separate *D. rivalis* from *D. amnicola*. Following the examination of type material from these two taxa we concluded that all features listed in the original descriptions are variable (including the pollinosity of the female parafacial, a feature we found to vary from entirely dusted to mostly glossy in the type series of *D. amnicola*), and that *D. rivalis* is a synonym of *D. amnicola*.

Furthermore, Huckett (1966, 1975) uses the strength of *av* bristles on the apical 1/2 of F2 to separate males *D. santamonicae* (strong and long) from *D. amnicola* (weak) and junior synonym *D. rivalis* (weak but slightly stronger). While the male holotypes of these taxa do exhibit a difference in the strength of the F2 *av*, we have found this feature to be variable. The configuration of the F2 *pv* row and the pollinosity of the thorax and abdomen appear to be more stable characters to separate the males of *D. santamonicae* from those of *D. amnicola* (and the similar *D. aldrichi*) (see couplet 21 of male key).

#### Drymeia aterrima

Taxon classificationAnimaliaDipteraMuscidae

(Wulp, 1896)

FD896114-DFA9-5B73-B564-54693D8FCBDD

Figs 1B , 12B

Pogonomyia aterrima Wulp, 1896: 335.

##### Type material examined.

None.

##### Other material examined.

More than 100 males and females: Neotropical: **Mexico**: Durango: 10 mi W. El Salto. (CNC).

##### Distribution.

Nearctic: USA (Washington, Montana, but see remark below). Neotropical: Mexico (Durango).

##### DNA Barcode.

None available.

##### Remarks.

This species was recently redescribed by Nihei and Carvalho (2004) but we are doubtful of the Nearctic records from Montana and Washington (USA). Not only do they create a highly disjunct distribution pattern for the species, they are based exclusively on female specimens which are similar to those of a number of other taxa including *Drymeia minor*, a species with a widespread distribution in the USA. Having examined numerous females associated with males of *D. aterrima* from El Salto, Mexico, we observed some differences with the description of Nihei and Carvalho (2004), mainly longer aristal hair (the longest 1.5–2.0 × as long as base) and the presence of 1 or 2 distinct *av* on the apical 1/3 of the mid femur (these reduced in a few specimens).

#### Drymeia cantabrigensis

Taxon classificationAnimaliaDipteraMuscidae

(Huckett, 1965)

7A369C2F-D040-50A9-9EAB-8FEEAEDC2C7D

Fig. 6A

Eupogonomyia cantabrigensis Huckett, 1965a: 300.

##### Type material examined.

*Eupogonomyia cantabrigensis* – ***Holotype*** male labelled “Cambridge Bay /N.W.T. 18.VII. 1950/ G. K. Sweatman”; “Type [red]”; “HOLOTYPE/ CNCNo. 8367 [red]”; “*Eupogonomyia/ cantabrigensis* Huck./ det. H.C. Huckett” (CNC). Allotype female labelled “Cambridge Bay /N.W.T. 20.VII. 1950/ E. H. N. Smith”; “Allo [red]”; “ALLOTYPE/ CNCNo. 8367 [red]”; “*Eupogonomyia*/ *cantabrigensis* Huck./ det. H.C. Huckett” (CNC). 3 ***paratypes*** males: “Cambridge Bay /N.W.T. 20.VII. 1950/ E. H. N. Smith”; “PARATYPE/No. 8367 [yellow]”; “*Eupogonomyia*/ *cantabrigensis* Huck./ det. H.C. Huckett” (CNC). 1 paratype male, same as previous but 18.VII. 1950. 2 paratypes males: “Cambridge Bay /N.W.T. 20.VII. 1950/ G. K. Sweatman”; “PARATYPE/No. 8367 [yellow]”; “*Eupogonomyia*/ *cantabrigensis* Huck./ det. H.C. Huckett” (CNC). 1 paratype male, same as previous but 21.VII.1950.

##### Other material examined.

More than 100 males and females: Nearctic: **Canada**: North West Territories: Arviat [formerly Eskimo point], Hooper Is., Mould Bay (Prince Patrick Is.), Sachs Harbour, Victoria Is.; Nunavut: Banks Is., Cambridge Bay, Kugluktuk, Masik Riv. (Banks I.); Yukon Territory: Herschel Is. (BUIC, CNC, LEM).

##### Distribution.

Nearctic: Canada (Northwest Territories, Nunavut, Yukon Territory).

##### DNA Barcode.

BOLDBIN: BOLD:AAD7664 (BIN merge with several other species, see Fig. 25). See Suppl. material 1: Table S1 for GenBank accession numbers.

##### Remarks.

While always lacking an anteroventral bristle on the midtibia, the females of this species are sometime indistinguishable from those of *D. setibasis* where this bristle is either present or absent. DNA barcodes for *D. cantabrigensis* were very similar and in some case identical to those of other species forming a cluster (or BIN merge) of seven named species (including *D. setibasis*) in BOLD:AAD7664 (Fig. 25). The males can be easily identified based on the distinctive chaetotaxy of the mid femur but since DNA barcodes do not discriminate between *D. cantabrigensis* and several other species including *D. setibasis*, the identification of females from these two species can be problematic in the Nearctic region.

#### Drymeia chillcotti

Taxon classificationAnimaliaDipteraMuscidae

(Huckett, 1965)

CC1063B5-09F2-519E-9E33-911640B8F59B

Fig. 3A

Bebryx chillcotti Huckett, 1965a: 302.

##### Type material examined.

*Bebryx chillcotti* – ***Holotype*** male labelled “Kidluit Bay, N./ Richards Is. W./ 27-VIII 1948. T./ J. R. Vockeroth”; “Type [red]”; “HOLOTYPE/ CNCNo. 8369 [red]”; “*Bebryx*/ *chillcotti* Huck./ det. H.C. Huckett” (CNC). Allotype female labelled “Chesterfiel/ N.W.T. 8.VIII. 1950/ J.G. Chillcott”; “Allo [red]”; “ALLOTYPE/ CNCNo. 8369 [red]”; “*Bebryx*/ *chillcotti* Huck./ det. H.C. Huckett” (CNC). Paratype male labelled “Kidluit Bay, N./ Richards Is. W./ 29-VIII 1948. T./ J. R. Vockeroth”; “*Bebryx*/ *chillcotti* Huck./ det. H.C. Huckett”; “PARATYPE/ No. 8369 [yellow]” (CNC). ***Paratype*** female labelled “Padlei N.W.T./ 6-VIII-1950/ R.A. Hennigar”; “Bebryx/ chillcotti Huck./ det. H.C. Huckett”; “PARATYPE/ No. 8369 [yellow]” (CNC).

##### Other material examined.

More than 180 males and females: Nearctic: **Canada**: British Columbia: Summit of Pink Mtn; Northwest Territories: 20 and 21 m. e. Tuktoyaktuk; Nunavut: Arviat [formerly Eskimo point], Char river nr Ranking Inlet, Clyde (Baffin Island), Coral Harbor (Southampton Island), Ford Lake, Landing Lake (7.5 km NW of Rankin inlet), Masik River (Banks Island.), Meliadine river, Padlei, Victoria Island; Yukon Territory: British Mts, Firth River, Richardson Mts, **USA**: Alaska: Noluk. Palaearctic: **Russia**: Taymyr Peninsula (BUIC, CNC, LEM, SZMN).

**Figure 3.**
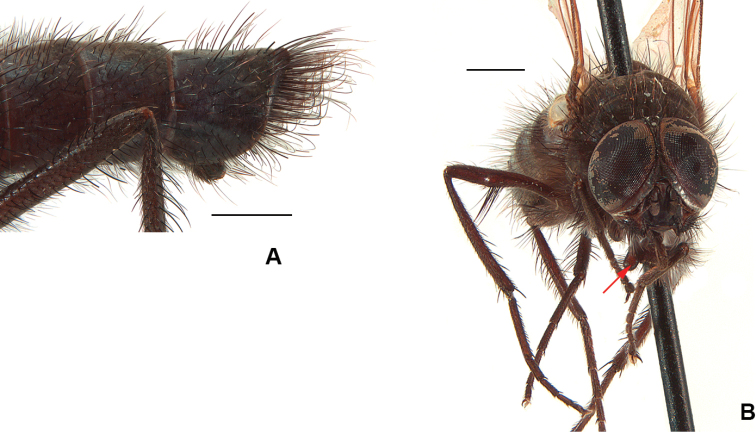
**A***D. chillcotti*, male abdomen **B***D. segnis*, male habitus, frontal view. Scale bars: 1 mm.

##### Distribution.

Nearctic: Canada (British Columbia, Northwest Territories, Nunavut, Yukon Territory), USA (Alaska). Palaearctic: Russia (Taymyr Peninsula).

##### DNA Barcode.

BOLDBIN: BOLD:ACA8934. See Suppl. material 1: Table S1 for GenBank accession numbers.

##### Remark.

DNA barcodes for material from Canada and Russia (Fig. 25) were very similar with a maximum intraspecific p-distance of 0.5%.

#### Drymeia firthiana

Taxon classificationAnimaliaDipteraMuscidae

(Huckett, 1965)

5451468B-8B8D-505C-AFD5-EBA73F93758C

Fig. 7A

Pogonomyia firthiana Huckett, 1965a: 298.

##### Type material examined.

*Pogonomyia firthiana* – ***Holotype*** male labelled “Firth River, Y.T./ 14-VII-1956/E.F. Cashman”; “Type [red]”; “Type/ HOLO/ No. 8366 [red]”; “*Pogonomyia*/ *firthiana* Huck./ det. H.C. Huckett” (CNC). Allotype female labelled “Firth River, Y.T./ 11-VII-1956/E.F. Cashman”; “Type [red]”; “Type/ Allo/ No. 8366 [red]”; “*Pogonomyia*/ *firthiana* Huck./ det. H.C. Huckett” (CNC). Paratype male labelled “Firth River, Y.T./ 11-VII-1956/R.E. Leech”; “PARATYPE/ No. 8366 [yellow]”; “*Pogonomyia*/ *firthiana* Huck./ det. H.C. Huckett” (CNC); ***Paratype*** female labelled “Firth River, Y.T./ 14-VII-1956/R.E. Leech”; “PARATYPE/ No. 8366 [yellow]”; “*Pogonomyia*/ *firthiana* Huck./ det. H.C. Huckett” (CNC).

##### Other material examined.

12 males and females: Nearctic: **Canada**: Yukon Territory: 14 km WSW Burwash Flats, Firth River; **USA**: Alaska: Schrader L. Palaeartic: **Kazakhstan**: Dzungarian Alatau, Sarkand River; **Russia**: Altai Republic, Khakasiya, Tyva (BUIC, CNC, SZMN).

##### Distribution.

Nearctic: Canada (Yukon Territory); USA (Alaska). Palaearctic: Russia (Altai Mts, Khakasiya, Tyva), Kazakhstan.

##### DNA Barcode.

BOLDBIN: BOLD:ADE2127. See Suppl. material 1: Table S1 for GenBank accession numbers.

##### Remark.

Only specimens from Russia were available for DNA barcoding (Fig. 25) with p-distances ranging from 0.0% to 0.16%.

#### Drymeia flavinervis

Taxon classificationAnimaliaDipteraMuscidae

(Malloch, 1915)

22F28D38-DC66-5E06-8B22-5DA4AC23193C

Fig. 9A

Pogonomyia flavinervis Malloch, 1915: 356.
Spilogaster nitens Stein, 1898: 199 [Junior primary homonym of Spilogaster nitens Macquart, 1855].
Pogonomyia flavipennis Stein, 1920: 21.

##### Type material examined.

*Pogonomyia flavinervis* – ***Lectotype*** male labelled “INHS/Insect Collection/ 238,900”; “N. Ill.”; “LECTOTYPE/ Pogonomyia/ flavinervis/ ♂ Malloch [red]” (INHS). ***Allotype*** female labelled “INHS/Insect Collection/238,901”; “Algonquin, Ill./ 5.24.95 -110”; “Lecto-/ALLOTYPE/ Pogonomyia flavinervis/ ♀ Malloch [blue]” (INHS).

##### Other material examined.

More than 100 males and females from: Nearctic: **Canada**: Alberta: Elkwater Park; Manitoba: Aweme, Bald Head Hills, 2 mi. E Douglas, 9 mi N. Forrest, Deloraine, Ninette, 30 mi N. Roblin, 3 mi. S. Shilo, 5 mi. SW Shilo, 2 mi. W Stockton, Teulon, Transcona, Turtle Mt., Virden; Nova Scotia: 4 km before Meat Cove (Victoria Co.); New Brunswick: Perth; Ontario: Bell’s Cor., Britannia, N. Burgess Twp., Burke Falls, Chatham, Constance L. (South March), Emo, Finland, 7 mi. E. Griffith, Hart L., Leamington, Metcalfe, Midland, Maynooth, March Twp., Marmora, Muskoka, Ojibway, Orillia, Ottawa, Point Pelee Ntl. Park, Pelee Is., Pinewood, Point Pelee, Rainy River, Rondeau Pr. Pk St. Lawrence Is. Nat. Park; Quebec: Alcove, Aylmer, Breackenridge; Gracefield, Laniel, Meach Lake, Mt. Auclair, Mt. Xalibu, Old Chelsea, Petit Mt St-Anne; Saskatchewan: Big River, Canora, Kenosee; **USA**: Maine: Tableland (Mt. Katahdin); Michigan: Isle Royale; Minnesota: Lake city; Virginia: Hawksbill (Shenandoah Ntl. Pk.) (BUIC, BIOUG, CNC).

##### Distribution.

Nearctic: Canada (Alberta to New Brunswick), USA (South Dakota, Wisconsin, Illinois to Maine).

##### DNA Barcode.

BOLDBIN: BOLD:ACA6790. See Suppl. material 1: Table S1 for GenBank accession numbers.

##### Remark.

DNA barcodes were available for material from Quebec and Ontario (Canada) with p-distances ranging from 0.0% to 0.16% (Fig. 25).

#### Drymeia glacialis

Taxon classificationAnimaliaDipteraMuscidae

(Rondani, 1866)

1CD22236-01D1-58DB-951E-F8F014DBE822

Figs 2H , 4E , 6E

Aspilia glacialis Rondani, 1866: 87.
Pogonomyia alpicola Rondani, 1871: 337. syn. nov.
Pogonomyia alpicola var. tundrica , Schnabl in Becker et al. 1915: 48.

##### Type material examined.

None.

##### Other material examined.

More than 100 males and females: Nearctic: **USA**: Colorado: Cameron Pass, Cottonwood Pass (Chaffee Co.), Echo Lake (Mt. Evans), Floral Park, Independance Pass (Lake Co.), Loveland Pass, Nederland, Summit Lake (Mt. Evans); Wyoming: Delaey Creek Park, Snowy Range Mts., Togwotee Pass (Teton Co.). Palaearctic: **Austria**: Hohe Tauern Nat. Pk., Igls, Obergurgl; **Italy**: Karthaus; **Mongolia**: Ara-Khangaiskii aimak, 7 km SW Taryata; **Russia**: Altai Republic, Khakasiya, Tyva; **Switzerland**: Julierpass (BUIC, CNC, SZMN).

**Figure 4.**
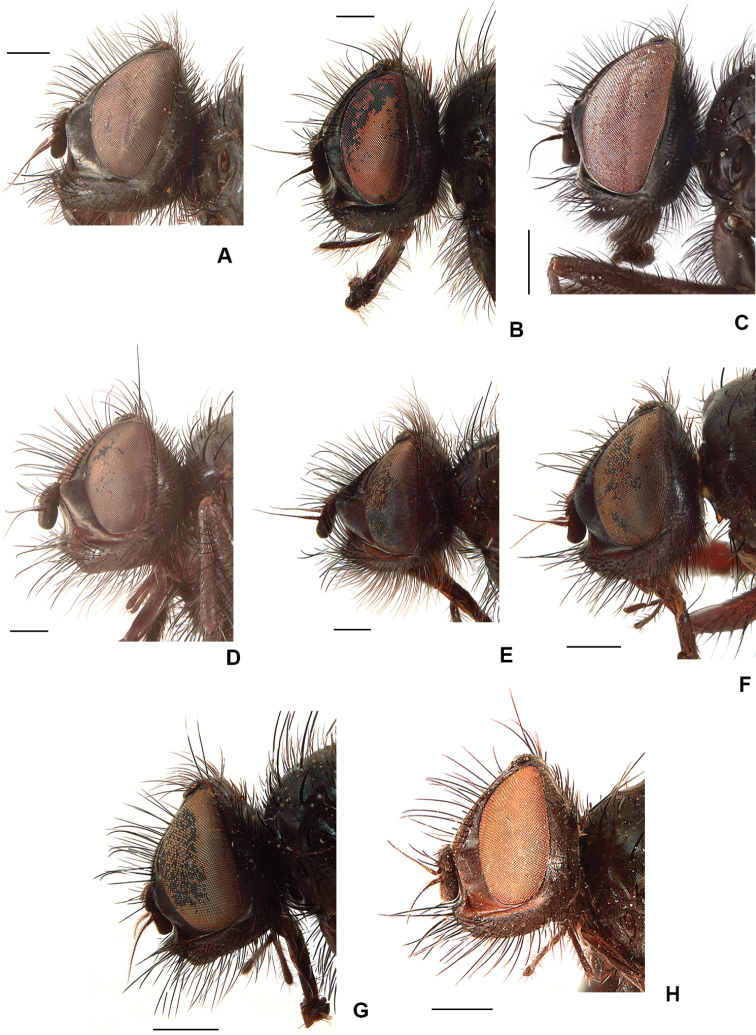
Male head, lateral view **A***D. pribilofensis***B***D. neoborealis***C***D. vockerothi***D***D. spinitarsis***E***D. glacialis***F***D. santamonicae***G***D. amnicola***H***D. minor*. Scale bars: 0.5 mm.

##### Distribution.

Nearctic: Canada (Alberta, Labrado,r and Newfoundland), USA (Rocky Mts down to New Mexico). Palaearctic: from Europe eastwards to the Far East of Russia.

##### DNA Barcode.

BOLDBIN: BOLD:AAC1021 (BIN merge with *D. quadrisetosa*). See Suppl. material 1: Table S1 for GenBank accession numbers.

##### Remarks.

We were recently informed that the holotype of *Aspilia glacialis* Rondani, previously considered lost (Hennig 1962b: 677; Pont 1986: 73), had been located in the Museo di Storia Naturale, Sezione di Zoologia ‘’La Specola’’, Università di Firenze (MZUF). While we did not examine this material ourselves, the specimen has been unambiguously recognised by A.C. Pont as *D. alpicola* (pers. comm.) and we consider his expertise sufficient to recognise that the earlier name of *D. glacialis* must be given precedence over *D. alpicola*. Additional details about this new synonymy will be published in an upcoming work (A.C. Pont, pers. comm.).

In the Nearctic region, females of this Holarctic species can be distinguished from those of *D. quadrisetosa* only by the slightly darker wing base. However, this colour character appears variable in the Palaearctic region where Russian material shows a darker wing base (congruent with Nearctic females) while females from a series we examined from Austria as well as the holotype of *D. glacialis* (A.C. Pont pers. comm.) display a pale wing base similar to that of *D. quadrisetosa*, a species known only from the Nearctic region.

DNA barcodes for *D. glacialis* (all from Russian specimens) were very similar to those of *D. quadrisetosa* (specimens from Russia and Canada), forming a cluster with p-distances ranging from 0.0% to 1.72% for BOLD:AAC1021 (Fig. 25). Males of these two species can be easily distinguished based on distinctive leg chaetotaxy (see key to males) but since DNA barcodes do not discriminate between the two species, the identification of females can be problematic in the Nearctic region, as differences in wing base colour between the two taxa can sometimes be very subtle, especially for material kept in ethanol for long periods.

#### Drymeia groenlandica

Taxon classificationAnimaliaDipteraMuscidae

(Lundbeck, 1901)

D0C5EEA3-F474-552A-A74C-3D481812E2E6

Fig. 2C

Ophyra groenlandica Lundbeck, 1901: 281.

##### Type material examined.

None.

##### Other material examined.

More than 100 males and females: Nearctic: **Canada**: Manitoba: Churchill, Wapusk Ntl. Pk; Northwest Territories: Tuktoyaktuk; Nunavut: Arviat [formerly Eskimo point], Axel Heiberg Island, Aulavik Nat. Pk, Baker Lake, Coral Harbour (Southampton Island), Clyde (Baffin Island), Ellesmere Island, Eureka (Ellesmere Island), Forsheim peninsula (Ellesmere Island), Hazen Camp (Ellesmere Island), Kugluktuk [formerly Coppermine], Lake Hazen (Ellesmere Island), Masik River (Banks Island), Padlei, Tranquary Fiord (Ellesmere Island); Yukon Territory: North Fork Crossing mi 42 peel plt. Rd, Ogilvie Mountains. **USA**: Alaska: Schrader L. **Greenland**: Nedre Midsommer Sö, Zackenberg. Palaearctic: **Russia**: Chukotka: Pevek, Taymyr Peninsula: Ary-Mas cordon, Sakha Republik: Chokurdakh, Indigirka River (BIOUG, BUIC, CNC, LEM, SZMN).

**Figure 5.**
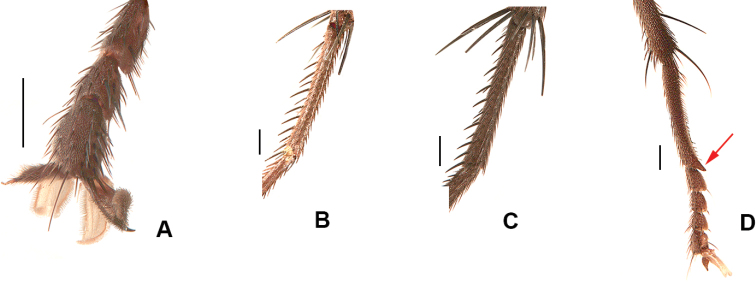
Tarsomeres **A***D. pribilofensis*, male fore tarsomeres 3–5 **B***D. spinitarsis*, male mid tarsomere 1 **C***D. spinitarsis*, female mid tarsomere 1 **D***D. quadrisetosa*, male fore tarsomeres 1–5. Scale bars: 0.25 mm.

**Figure 6.**
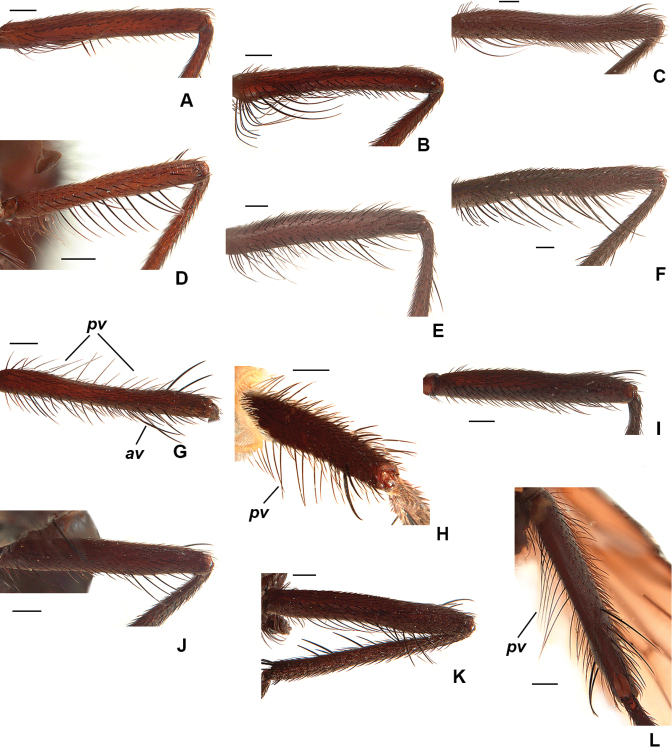
Male mid femur, anterior view (except **G, H, L**) **A***D. cantabrigensis***B***D. setibasis***C***D. vockerothi***D***D. profrontalis***E***D. glacialis***F***D. quadrisetosa***G***D. santamonicae*, antero-dorsal view **H***D. amnicola*, postero-dorsal view **I***D. similis***J***D. amnicola***K***D. hucketti***L***D. aldrichi*, ventral view. Scale bars: 0.25 mm.

##### Distribution.

Nearctic: Canada (Manitoba, Northwest Territories, Nunavut, Yukon Territory), USA (Alaska), Greenland. Palaearctic: Russia (Chukotka, Taymyr Peninsula, Sakha Republic).

##### DNA Barcode.

BOLDBIN: BOLD:AAL9801. See Suppl. material 1: Table S1 for GenBank accession numbers.

##### Remark.

DNA barcodes for material from Canada, Greenland, and Russia (Fig. 25) were similar with a maximum intraspecific p-distance of 0.76%.

#### Drymeia hucketti sp. nov.

Taxon classificationAnimaliaDipteraMuscidae

9D4F567B-220B-56D3-955F-A17FB8115C8E

http://zoobank.org/FCA7C9F9-E170-4DCD-A7DB-3DC0A14D0DA4

Figs 2I , 6K , 7B , 11E , 12A , 16 , 17 , 18

##### Type material.

***Holotype*** male labelled “Wood Mountain/ Sask 17.6.1955/ A.R. Brooks”; “HOLOTYPE/ Drymeia hucketti ♂/ Savage & Sorokina [red]” (CNC). ***Paratypes***: all with “PARATYPE/ Drymeia hucketti/ Savage & Sorokina [yellow]” (CNC unless otherwise indicated): 1 male, same as holotype. 1 male, same as holotype except (BUIC) 1 male and 1 female labelled “Scout Lake/ Sask. 17.VI.1955/ 49°20', 106°0'/ J.R. Vockeroth”. 1 male labelled “Val Marie, Sask./ 49°15', 107°44'/ 12.VI.1955. 1 female same as previous but 9.VI.1955. 1 female labelled “Elkwater L.,/ Alta, 10.VI-1956/ E.E. Sterns” (BUIC). 1 female labelled “Elkwater Alta/ 8.VI. 1952/ A.R. Brooks”. 1 male labelled “Elkwater park, Alta/ 31.V. 1952/ L. A. Konotopetz”. 1 male same as previous but 29.V.1952. 1 female labelled “Banff, Alta./ 4.VII.1924/ Eric Hearle”. 1 female labelled “12 mi. N. of Banff/ Banff-Jasper Hw./ 4500' 26-VII-55/ R. Coyles”. 1 female labelled “Manyberries, Alta/ 4-VI-.1956/ E.E. Sterns”. 1 male “Manyberries/ Alta. 6.VI 1955/ A.R. Brooks”. 1 female labelled “Highwood Summit/ Kananasksis-Coleman/ Hwy., Alta, 72-8000'/ 14-VIII-1955/ J.R. McGillis”. 1 male and 1 female labelled “Waterton, ALTA/ 11 June 1962/ K.C. Hermann”. 1 male labelled “BIOUG04930-G08/ CAN: AB: Waterton Lakes NP; Red/ Rock Parkway moraine/ grassland 49.0813°N -113.8792°W/ 1335 m as IBIOBus 2012 6/27/2012” (BIOUG). 1 female same as previous except BUIOUG05064-E03. 1 female same as previous except BIOUG05064-D12. 1 female same as previous except BIOUG05064-D11. 1 female same as previous except BIOUG05064-B07. 1 female same as previous except BIOUG05064-D03 and (BUIC). 1 male same as previous except BIOUG05213-G11 and 6/24/2012. 1 male labelled “BIOUG08066-D02/ CAN: BC, 10 km W Kamloops; New/ Alton Mine Grassland Protected/ Area (control side) – Site 4/ 50.655°N, 120.655°W Chrystal/ Simon 6/13/2013” (BIOUG). 1 female same as previous except BUIOUG07269-F05. 1 male same as previous except BIOUG08066-F06 and (BUIC). 1 male labelled “Mt. Lolo/ Kamloops, B.C./ 2.VI.1938/ G.S. Walley”. 1 female labelled “Moosehorn L.,/ B.C. 28.VII 1960/ 58°10', 132°07'/ W.W. Moss 4500'”. 1 male same as previous except 27.VII.1960, R. Pilfrey. 1 male labelled “Chilcothin/ 5/27/29 BC”. 1 male and 1 female labelled “B.C. Anarchist/ Mt. 21.VI.1982/ B.V. Peterson”. 1 female labelled “BIOUG03134-E09/ CAN: SK; Grasslands National Park;/ just past bridge over Frenchman/ River 49.1494°N, 107.5302°W R./ Sissons 06/05/2012 to 06/12/2012” (BIOUG). 1 female labelled “BIOUG51024-D11/ USA: MT; Missoula County/ Florence MPG Ranch – Site 3/ 46.6905°N, 114.0265°W 1136m 13-/16 May 2019 Mat Seidensticker” (BIOUG). 1 female, same as previous except BIOUG24024-E02. 1 female, same as previous except BIOUG24024-E07. 1 male, same as previous except BIOUG24024-E03. 1 male, same as previous except BIOUG24024-E10. 1 male, same as previous except BIOUG24024-F01.

**Figure 7.**
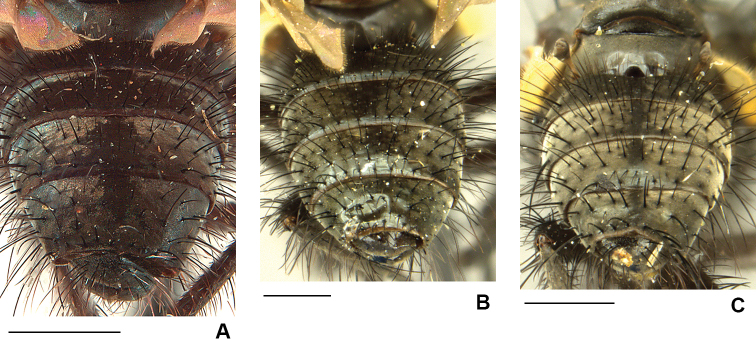
Male abdomen **A***D. firthiana***B***D. hucketti***C***D. minor*. Scale bars: 1 mm.

##### Other material examined.

2 males: Nearctic: **Canada**: Alberta: Banff National Park; Saskatchewan: Grasslands National Park.

##### Etymology.

The species name is a patronym in honour of Hugh C. Huckett, a major contributor to the study of Nearctic Muscidae.

##### Diagnosis.

Small glossy black species with a long, strong prealar, lower margin of face projecting slightly beyond lower level of frons (Fig. 16A, 18A), and 2+3 *dc.* Male F2 with *av* row strong and regular, covering apical 2/3 to 3/4, and with *pv* row long and strong, at least 2 × as long as width of femur on apical 1/2, and T3 with a short but distinct ventral apical process (Fig. 16D). This species is similar to *Drymeia minor* (Malloch, 1918) but can be distinguished from it in the female by the presence of a large undusted glossy patch on the parafacial near the base of the antenna (Fig. 18B) and, in both sexes, by the projecting face, broader ventral margin of parafacial, pubescence pattern of arista and mostly shiny abdomen. Females are also similar to those of *D. amnicola* (see couplet 14 of female identification key) but these taxa have very different distribution ranges.

##### Description.

**Male.** Body length: 4.6–6.6 mm; wing length 4.1–5.1 mm.

***Head*:** Ground colour black; eye bare; fronto-orbital plate and parafacial silvery pruinose; face grey, gena and lower occiput grey pruinose; fronto-orbital plates touching in the middle; frons at narrowest point 2–3 × as wide as width of anterior ocellus; parafacial in lateral view with ventral margin broader than width of first flagellomere; lower margin of face projecting slightly beyond lower level of frons (Fig. 16A); gena at narrowest point 1.3 × length of first flagellomere, densely setulose and with a group of upcurved setae on anterior part of genal dilation; 11–13 frontal setae (including interstitials) reaching to anterior ocellus; antenna black; first flagellomere 1.2 × as long as wide; arista with hair much denser on basal 1/2 (longest hair as long as basal diameter of arista) and usually with sparse and very dorsal short hair on apical 1/2; palpus black; proboscis elongate with prementum much longer than palpus, mostly undusted and shiny; labella moderately developed.

**Figure 8.**
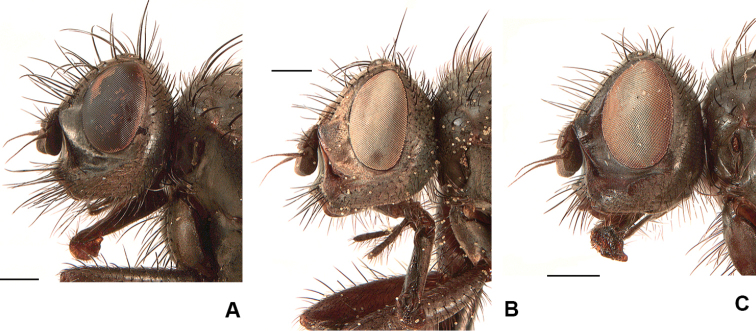
Female heads **A***D. spinitarsis***B***D. pribilofensis***C***D. setibasis*. Scale bars: 0.5 mm.

***Thorax*:** Ground colour black; scutum, postpronotal lobe, notopleuron, postalar callus and pleuron subshiny and light grey dusted; anepimeron and katepimeron bare; notopleuron densely setulose; 2+3 *dc*; prealar long and strong.

***Legs*:** Black; T1 with 1–3 *pv*; F2 with *av* row strong and regular, slightly longer than width of femur, covering apical 2/3 to 3/4 and with bristles curved on basal 1/2 (Fig. 16C), 2 or 3 preapical *pd-p*, with complete row of long strong *pv*, at least 2 × as long as width of femur on apical 1/2; T2 usually without *av* (one paratype with 1 short *av* on one side), 3–6 *pd*, 2–4 *pv* (some specimens also with 2 or 3 *p*); F3 with *av* row stronger on apical 1/3, without *pv* except one hair near base; T3 with 4 or 5 *av*, 6 or 7 *ad*, 4 or 5 *pd*, 4 or 5 short hair-like *pv* in middle part, ventral apical process short but distinct (Fig. 16D), apical *pv* distinct but no longer than 1/2 the length of apical *av*.

***Wing*:** Brown, darker near base; basicosta and tegula black; costal spinules weak and costal spine reduced; calypters with membrane and edges yellow.

***Abdomen*:** Conical, ground colour black; lightly grey dusted and mostly shiny, tergites without distinct dark central vittae (Fig. 16B); sternite I bare; sternite V as in Fig. 17C.

***Terminalia*:** Fig. 17A, B.

**Female.** Body length: 5.7–6.5 mm; wing length: 4.6–5.0 mm (Fig. 18A). Differs from the male as follows:

***Head*:** Frontal triangle undefined; ocellar triangle mostly glossy; frontal vitta black, deep brownish dusted; parafacial with large undusted shiny patch near base of antenna reaching up to or almost up to eye (Fig. 18B); frons at midpoint approximately 0.35 × as wide as head and approximately 1.2 × as long as wide; fronto-orbital plate narrow, approximately as wide as distance between inner margins of posterior ocelli; 3–6 medioclinate frontal setae and several weaker interstitials, two short reclinate and lateroclinate orbital setae followed by one (occasionally two) stronger proclinate orbital seta; arista as in male (Fig. 18C).

***Thorax*:** As in male.

***Legs*** (chaetotaxy described in full): T1 with 2 pv; F2 variable with 1–6 *av* (most specimens with 4 or 5), approximately as long as width of femur, and no *pv*; T2 with 2 or 3 *ad*, 3 or 4 *pd*, 2 *pv*; T3 with 3–5 *av*, 3–6 *ad*, 4 *pd*, and usually without *pv* (if present then very weak and short), apical *pv* distinct, at least 1/2 as long as apical *av*.

***Wing*:** Veins yellow at least near base, membrane deep yellow near base, the remainder pale yellow to pale brown.

***Abdomen*:** With little to no dusting, shiny.

##### Distribution.

Nearctic: Canada (Alberta, British Columbia, Saskatchewan), USA (Montana).

##### DNA Barcode.

BOLDBIN: BOLD:ACA9214. See Suppl. material 1: Table S1 for GenBank accession numbers.

##### Remarks.

The discovery of this species resulted from an exploration of all public *Drymeia* COI sequences found in BOLD (> 2800) which brought our attention to BOLD:ACA9214, a BIN including several well-preserved undetermined specimens of both sexes which turned out to be morphologically distinctive from any other species previously known to us. DNA barcodes for material from Canada and the United States (Fig. 25) were available with intraspecific p-distances ranging from 0.0% of 0.35%.

#### Drymeia latifrons

Taxon classificationAnimaliaDipteraMuscidae

(Malloch, 1918)

E970512F-F0BA-5815-BDA0-80CA865ADD6B

Fig. 11B

Pogonomyia latifrons Malloch, 1918: 281.

##### Type material examined.

*Pogonomyia latifrons* – ***Holotype*** female labelled “INHS/ Insect Collection/ 238,902”; “Tenn. Pass. Colo./Jul.24' 17–JMA”; “TYPE/Pogonomyia/ latifrons/ ♀ Malloch [red]”; “Pogonomyia/ latifrons/ Mall. Type” (INHS).

##### Other material examined.

None.

##### Distribution.

Nearctic: USA (Colorado).

##### DNA Barcode.

None available.

##### Remarks.

The female holotype, with its unique combination of a broad frons, mid femur with weak *av* setae on apical 1/2, mid tibia with a single strong *av*, hind tibia with a strong apical *pv*, and wing membrane pale brown, is distinctive from any other specimen we have examined.

**Figure 9.**
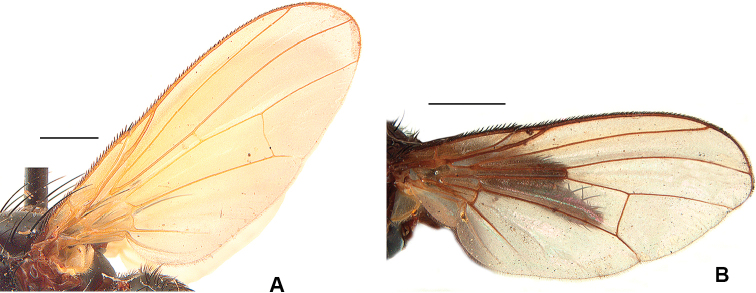
Female wings **A***D. flavinervis***B***D. aldrichi*. Scale bars: 1 mm.

#### Drymeia minor

Taxon classificationAnimaliaDipteraMuscidae

(Malloch, 1918)

7115BA07-E0C4-51AD-8B22-6F136A869DB2

**Figure 10.**
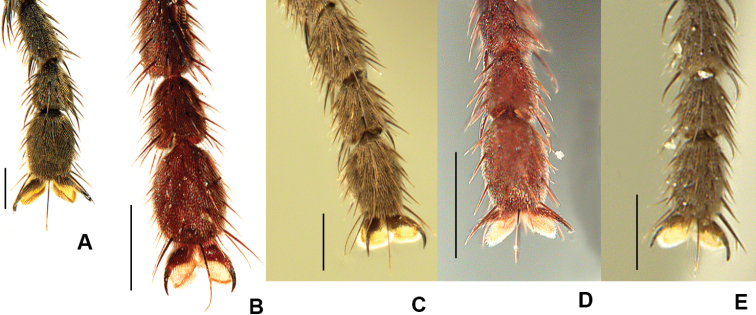
Female fore tarsomeres 3–5 **A***D. ponti***B***D. aldrichi***C***D. similis***D***D. profrontalis***E***D. minor*. Scale bars: 0.25 mm.

Figs 4H , 7C , 10E , 12C

Pogonomyia minor Malloch, 1918: 280.

##### Type material examined.

*Pogonomyia minor* – ***Holotype*** male labelled “Top of Las/ Vegas Range/VI.28.02 NM”; “HoloTYPE/6201 [red]”; “HoloTYPE/ Pogonomyia/ MINOR/ Mall. [red]” (ANSP).

##### Other material examined.

9 males, 3 females: Nearctic: **USA**: Colorado: Gunnison, Mt. Evans (CNC, BUIC).

##### Distribution.

Nearctic: Canada (British Columbia, Alberta, Saskatchewan but see remark below); USA (California, Colorado, New Mexico, Oregon, Wyoming) (but see Remarks).

##### DNA Barcode.

BOLDBIN: BOLD:ADZ5293. See Suppl. material 1: Table S1 for GenBank accession numbers.

##### Remarks.

Based on the distribution of examined specimens (including the holotype of *D. minor*) as well as on the distribution of all public sequences for BOLD:ADZ5293 (*D. minor*) and BOLD:ACA9214 (*D. hucketti* sp. nov.), we suspect that previously published Canadian records of *D. minor* may actually belong to *D. hucketti* sp. nov. but additional data will be necessary for confirmation. Only specimens from Colorado (USA) were available for DNA barcoding (Fig. 25) with p-distances ranging from 0.0% to 0.47%

#### Drymeia neoborealis

Taxon classificationAnimaliaDipteraMuscidae

(Snyder, 1949)

5F0FA94C-01F4-5650-BDD2-30005361152C

Fig. 1C

Aricia borealis Malloch, 1919: 64.
Helina neoborealis Snyder, 1949: 122 [replacement name for Aricia borealis Malloch, 1919].

##### Type material examined.

*Aricia borealis* – ***Holotype*** male labelled “Bernard/ Harbour/ N.W.T./ July”; Canadian/ Arctic/ Expedition/ F. d. 1916”; TYPE/ H. borealis/ Mall./ No. 1176 [red]”/ “374” (CNC). ***Allotype*** [belongs to *Spilogona tundrae* Schnabl] female labelled “Helina/ PARATYPE/ *borealis*/ Mall/ No 1176 [yellow]”/ Cape Bathurst/ N. W. T.”; “Arctic/ Expedition/ July 26 191[no last digit]”; “F. Johansen/ Coll.”; “387”; “*Spilogona*/ *tundrae* Schn./ det. V/ Sorokina, 2019” (CNC).

##### Other material examined.

More than 50 males and females: Nearctic: **Canada**: Northwest Territories: Banks Island, Masik River, Victoria Island; Nunavut: Aulavik, Baker Lake, Cambridge bay, Char river nr Ranking Inlet, Chesterfield, Landing Lake (7.5 km NW of Rankin inlet). Palaearctic: **Russia**: Chukotka AO: Wrangel Island (BUIC, CNC, SZMN).

##### Distribution.

Nearctic: Canada (Northwest Territories, Nunavut); USA (Alaska, California, Colorado). Palaearctic: **Russia** (Wrangel Island).

##### DNA Barcode.

BOLDBIN: BOLD:ACA8935. See Suppl. material 1: Table S1 for GenBank accession numbers.

##### Remarks.

Huckett (1975: 103) mentions an undescribed *Eupogonomyia* species from California (female only) that would be distinguished from *D. neoborealis* based on the complete absence of a prealar bristle (the prealar is short but often visible in *D. neoborealis*) as well as projecting oral margins. We have found these features to be variable in the material we have examined (including specimens with DNA barcodes) and conclude that the unnamed species mentioned by Huckett (1975) falls within the range of known variations for *D. neoborealis*. While the COI sequence from the only Russian specimen in our data set showed a minimum intraspecific p-distance of 1.37% with the Canadian specimens, all DNA barcodes for *D. neoborealis* clustered together in BINBOLD:ACA8935 (Fig. 25) with a maximum intraspecific p-distance of 1.52%.

#### Drymeia ponti sp. nov.

Taxon classificationAnimaliaDipteraMuscidae

AF631217-181A-557E-824C-D12BEDF83754

http://zoobank.org/DCDED5B3-721C-442C-A16C-590AD57F5D4D

**Figure 11.**
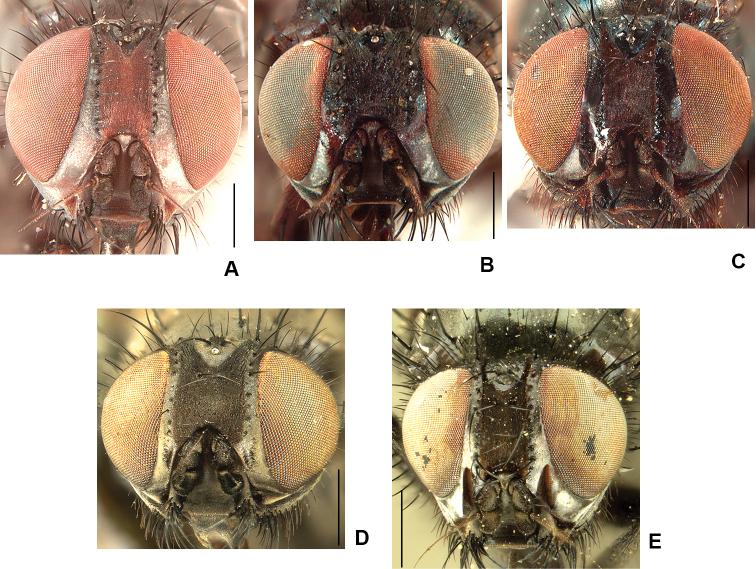
Female frons **A***D. similis***B***D. latifrons***C***D. profrontalis***D***D. ponti***E***D. hucketti*. Scale bars: 0.5 mm.

Figs 1A , 2A , 10A , 11D , 13 , 14 , 15

##### Type material.

***Holotype*** male labelled “W. Side Cortes Pass/ 1100' Mexico, Mexico/ 13-VIII-1954/ J.G. Chillcott”; “HOLOTYPE/ Drymeia ponti ♂/ Savage & Sorokina [red]” (CNC). ***Paratypes***: all with “PARATYPE/ Drymeia ponti/ Savage & Sorokina [yellow]”: 2 males and 15 females, same as holotype. 1 male and 2 females, same as holotype except (BUIC). 1 male and 5 females labelled “Rio Frio/ Fed. Dist. Of Mex./ I.IX.69/ D. Kritsch” (CNC). 1 male, same as previous except (BUIC). 1 male labelled “Rio Frio, MEX./ MWX.,27-VIII-69/ D. Kritsch BL 10,000'” (CNC).

##### Etymology.

The species name is a patronym in honour of Adrian C. Pont (UK), an exceptional dipterist and mentor to both co-authors.

**Figure 12.**
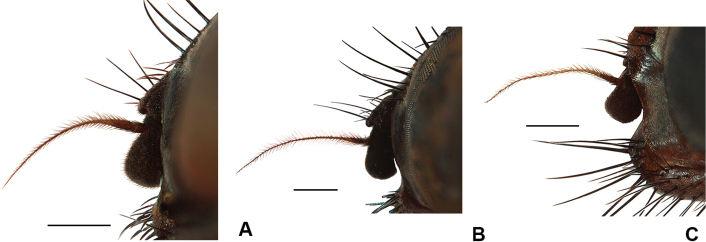
Female arista **A***D. hucketti***B***D. aterrima***C***D. minor*. Scale bars: 0.25 mm.

##### Diagnosis.

**Figure 13.**
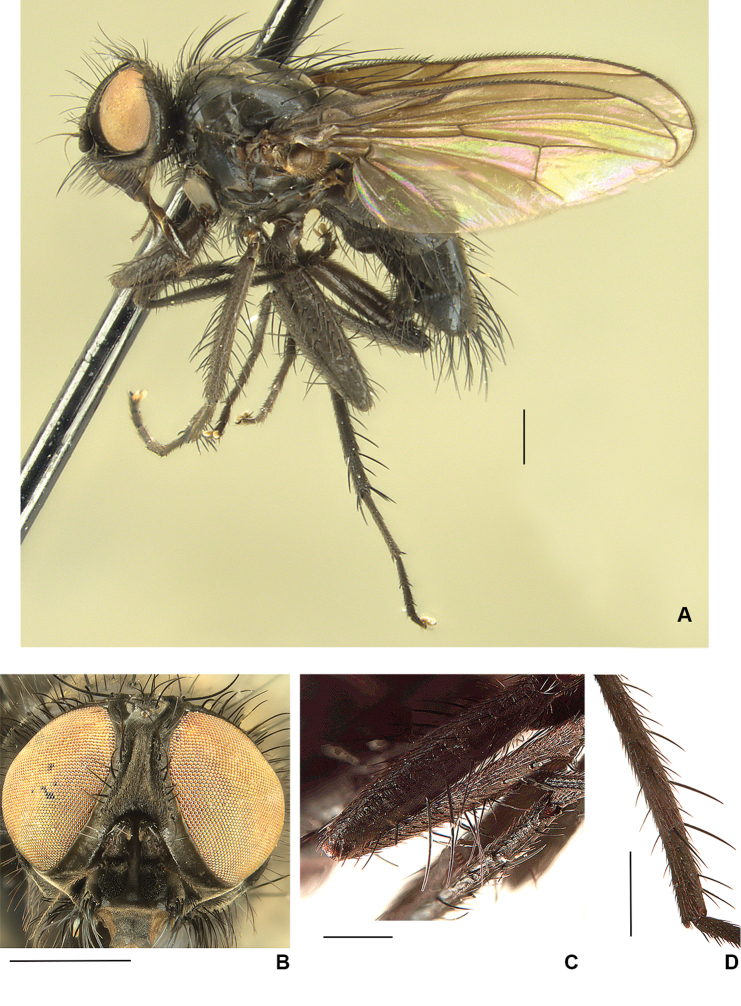
*Drymeia ponti* sp. nov. **A** male habitus, paratype **B** male head, frontal view **C** male mid femur, anterodorsal view **D** male hind tibia, anterior view. Scale bars: 1 mm (**A, B**); 0.5 mm (**C, D**).

Small dark species with strong prealar, 2+3 *dc* and strong costal spine. This species is similar to *Drymeia aterrima* (Wulp, 1896), especially in the dark male calypter, but can be distinguished from it in the male by a broad frontal vitta (Fig. 13B) and a distinct ventral apical process on T3 (Fig. 13D), and in the female by the presence of a flattened fore tarsomere 5 (Fig. 15C).

##### Description.

**Male.** Body length: 4.1–5.7 mm; wing length: 3.8–5.2 mm.

***Head*:** Ground colour black; eye bare; fronto-orbital plate and parafacial dark brown pruinose; face black, gena and lower occiput dark brown pruinose; frons at narrowest point approximately 1.5 × width of ocellar triangle with black frontal vitta exposed (Fig. 13B); parafacial in lateral view equal to or slightly wider than width of first flagellomere along most of its length (Fig. 13A); lower margin of the face projecting slightly beyond lower level of frons in lateral view; gena at narrowest point as high as length of first flagellomere, densely setose and without a group of upcurved setae on anterior part of genal dilation; 9–12 frontal setae (including interstitials) reaching to anterior ocellus; antenna black; first flagellomere 1.2 × as long as wide; arista swollen near base and pubescent, with longest hair as long as basal diameter of arista; palpus black; proboscis long and narrow with prementum approximately 2 × as long as palpus, undusted and glossy; labella small.

***Thorax*:** Ground colour black; scutum, postpronotum, notopleuron, postalar callus and scutellum with dense brown dusting; pleuron dark brown, slightly shiny with brown dusting; katepisternum with small anterior undusted glossy patch, meron entirely dusted; anepimeron and katepimeron bare; notopleuron setulose; *acr* 0+1; 2+3 *dc*; prealar long and strong, as long as second notopleural.

***Legs*:** Black; T1 with 2 *pv* on apical 1/2; F2 straight, with matching rows of long strong *av* and *pv* on apical 2/3, these much longer than width of femur (Fig. 13A, C), a complete row of short *ad*, and 2 or 3 preapical *pd-p*; T2 with 1 long and 2 shorter *ad* on apical 1/2, 4 *pd* and 1 *pv*; F3 with a complete row of *ad*, a row of *av*, longer on apical 1/2, a row of *p* on basal 2/3 and a row of long strong *pv* on apical 1/3; T3 with 2 *av*, 4 *ad*, 3 *pd* and with 4 short delicate *pv* on apical 1/2, ventral apical process short but distinct (Fig. 13D), apical *pv* absent or reduced, no longer than 1/2 the length of apical *av* when visible.

***Wing*:** Brown, darker at base with dark brown veins; basicosta and tegula black; costal spinules strong, with costal spine 2 × as long as costal spinules; calypters with membrane and edges dark brown.

**Figure 14.**
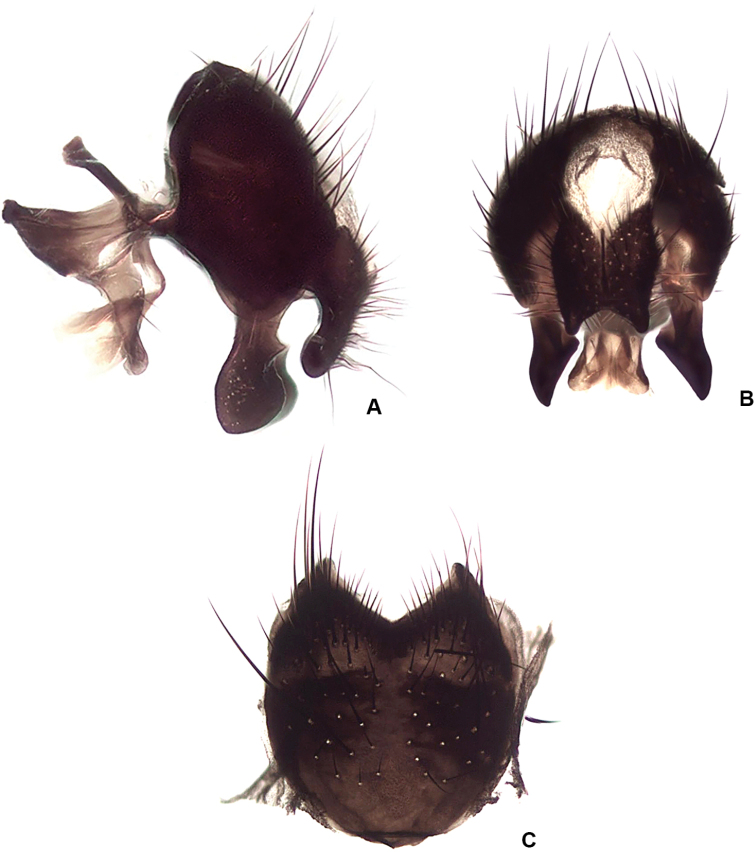
*Drymeia ponti* sp. nov., male terminalia **A** lateral view **B** posterior view **C** sternite V.

***Abdomen*:** Conical; ground colour black; brown dusted, subshiny with no median vittae; sternite I bare; sternite V as in Fig. 14C.

***Terminalia*:** Fig. 14A, B.

**Figure 15.**
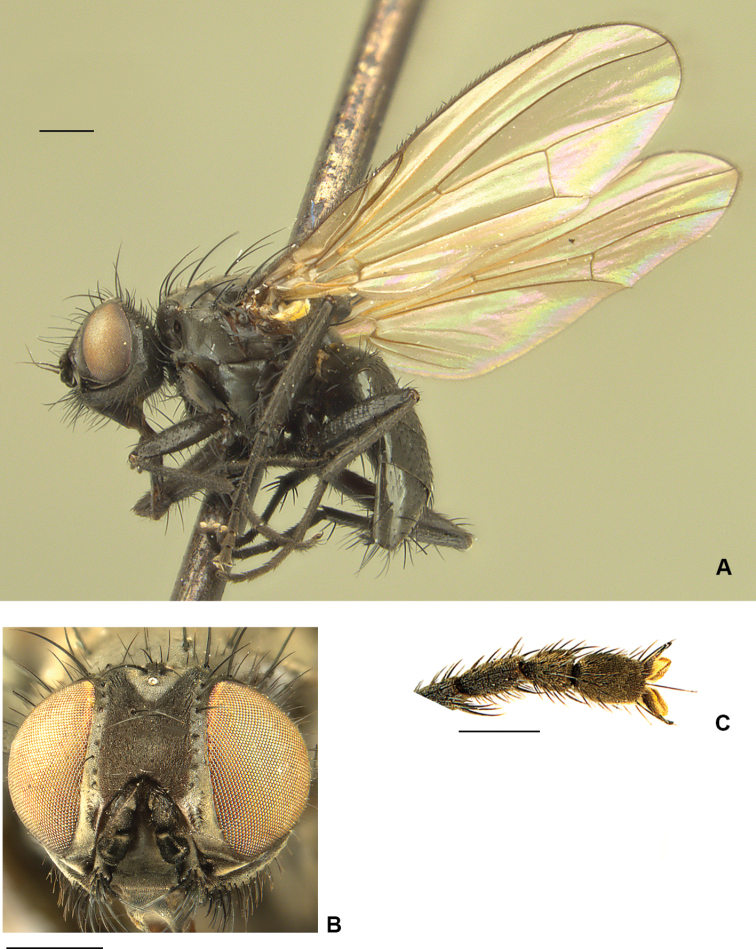
*Drymeia ponti* sp. nov. **A** female habitus, paratype **B** female head, frontal view **C** female tarsomeres 3–5. Scale bars: 1 mm (**A, B**); 0.5 mm (**C**).

**Female.** Body length: 4.5–6.5 mm; wing length: 4.0–5.5 mm. Differs from the male as follows:

***Head*:** Frontal triangle undefined; frontal vitta black; parafacial mostly dusted, with a small narrow shiny patch near base of antenna (Fig. 15A, B); frons at midpoint approximately 0.4 × as wide as head and approximately 0.9 × as long as wide; fronto-orbital plate narrow, approximately as wide as distance between inner margins of posterior ocelli; three or four medioclinate frontal setae and several weaker interstitials, three orbital setae, the upper two reclinate and lateroclinate, the lower one proclinate.

***Thorax*:** As in male.

***Legs*** (chaetotaxy described in full): T1 with 1 or 2 *pv*; fore tarsomere 5 distinctively flattened (Fig. 10A, 15C); F2 with 1 or 2 prebasal and 2 or 3 preapical *av* without *pv*; T2 with 0 or 1 *av* (most without), 2 or 3 *ad*, 4 or 5 *pd* and 2 *pv*; F3 with *av* row complete, without *pv*; T3 with 2 or 3 *av*, 3 or 4 *ad* and 3 *pd*, apical *pv* usually absent but if present, then no longer than 1/2 length of apical *av*.

**Figure 16.**
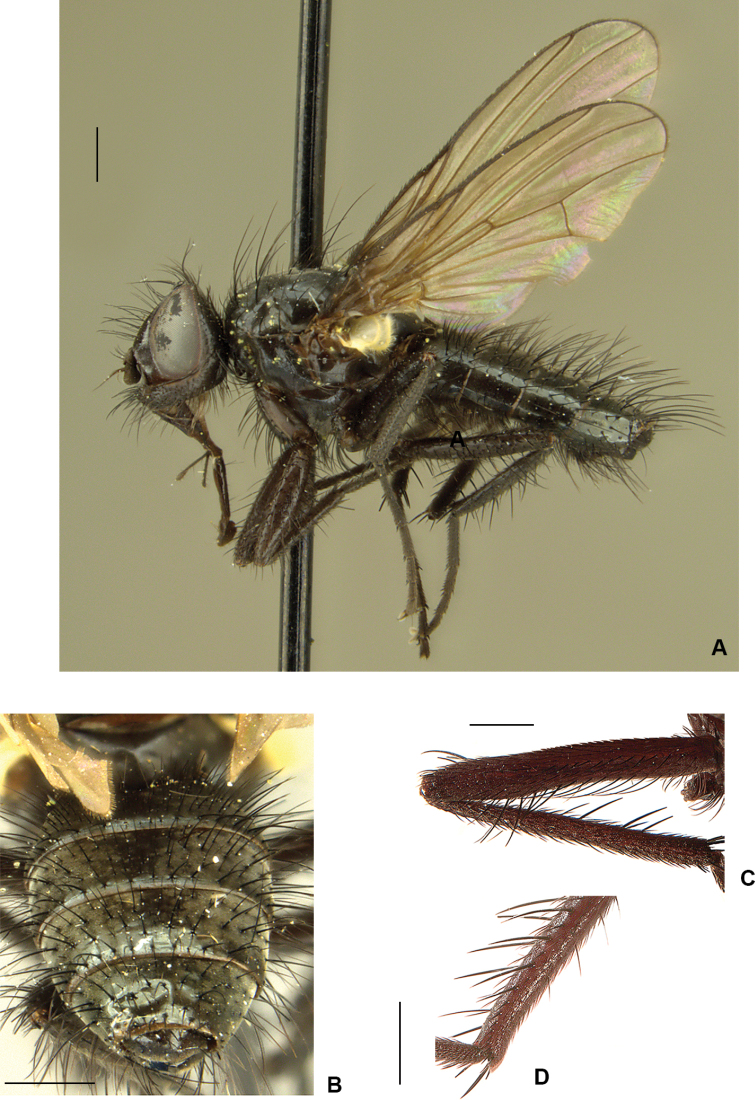
*Drymeia hucketti* sp. nov. **A** holotype habitus **B** male abdomen, dorsal view **C** male mid femur, anterior view **D** male hind tibia. Scale bars: 1 mm (**A, B**); 0.5 mm (**C, D**).

***Wing*:** Light brown, darker near base; with membrane and edges dark yellow.

***Abdomen*:** as in male.

##### Distribution.

Neotropical: Mexico (Mexico).

##### DNA Barcode.

None available.

#### Drymeia pribilofensis

Taxon classificationAnimaliaDipteraMuscidae

(Malloch, 1919)

9B290594-E2E3-5830-ADB3-CCAC8898D007

Figs 4A , 5A , 8B

Eupogonomyia pribilofensis Malloch, 1921: 179.
Pogonomyia inaequalis Malloch, 1922: 81.

##### Type material examined.

*Eupogonomyia pribilofensis* – ***Holotype*** male labelled “St. Paul Isd./ Alaska/ VI-21-20”; “Presented by/ G.D. Hanna/ Collector”/ “HOLOTYPE/ pribilifensis [red]”; “Eupogonomyia/ pribilofensis/ Mall. Type” (CAS).

##### Other material examined.

More than 400 males and females from: Nearctic: **Canada**: Manitoba: Churchill; Northwest Territories: Aklavik, Aulavik Nt. Pk. (Banks Island), Kidluit Bay, Mould Bay, Tuktoyaktuk; Nunavut: Arviat [formerly Eskimo point], Bathurst Inlet, Cambridge Bay, Chesterfield, Coral Harbour, Frobisher Bay, Kugluktuk [formerly Coppermine], Landing Lake (7.5 km NW of Rankin Inlet), Naujaat [formerly Repulse Bay], Padlei, Williamson Lake; Quebec: Inukjuak [formerly Port Harrison], Kangirsuk [formerly Payne Bay], Sugluk; Yukon Territory: Herschel Island. Palaearctic: **Russia**: Taymyr Peninsula: Ary-Mas cordon, 90 km NW Khatanga, Dixon; Yamalo-Nenez AO; Chukotka AO: Wrangel Island; Sakha Republic: Chokurdakh, Indigirka River (BUIC, CNC, LEM, SZMN).

##### Distribution.

Nearctic: Canada (Manitoba, Northwest Territories, Nunavut, Quebec, Yukon Territory), USA (Alaska). Palaearctic: Russia (W Siberia, Far East (Wrangel I.)).

**Figure 17.**
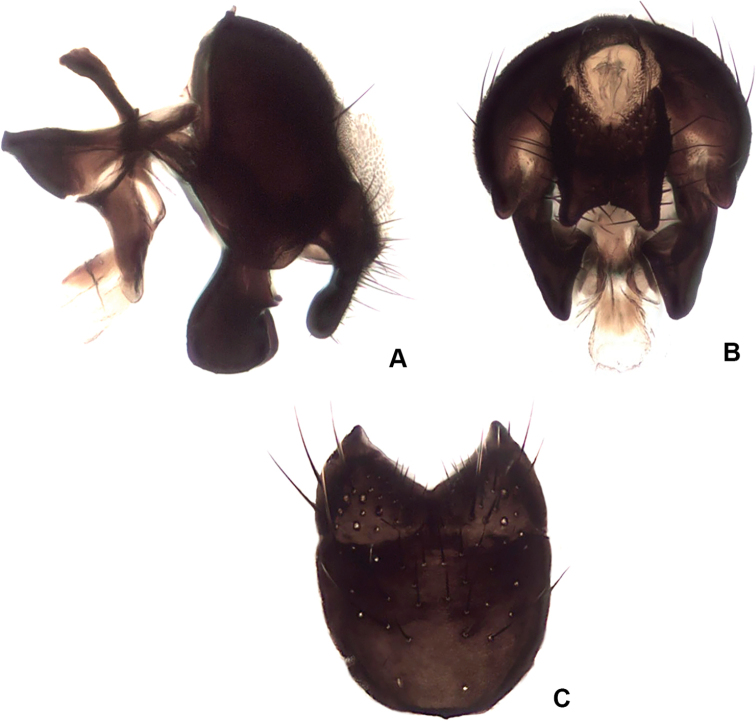
*Drymeia hucketti* sp. nov., male terminalia **A** lateral view **B** posterior view **C** sternite V.

##### DNA Barcode.

BOLDBIN: BOLD:AAD7664 (BIN merge with several other species, see Fig. 25). See Suppl. material 1: Table S1 for GenBank accession numbers.

##### Remark.

Males of this species can be readily identified based on the combination of a reduced prealar, strongly projecting face (Fig. 4A) and distinctive chaetotaxy of the mid femur but the females can be difficult to distinguish from those of *D. setibasis* and *D. cantabrigensis* (see key to females). All barcoded specimens of *D. pribilofensis* from our dataset clustered closest to one another on the neighbour-joining tree (Fig. 25) with a maximum intraspecific p-distance of 0.15% but the distance to *D. cristata*, one of the seven species found in the BOLD:AAD7664BIN merge was very low (min p-distance = 0.61%). We would therefore not recommend using COI to discriminate specimens of *D. pribilofensis* from those of other species in this BIN.

**Figure 18.**
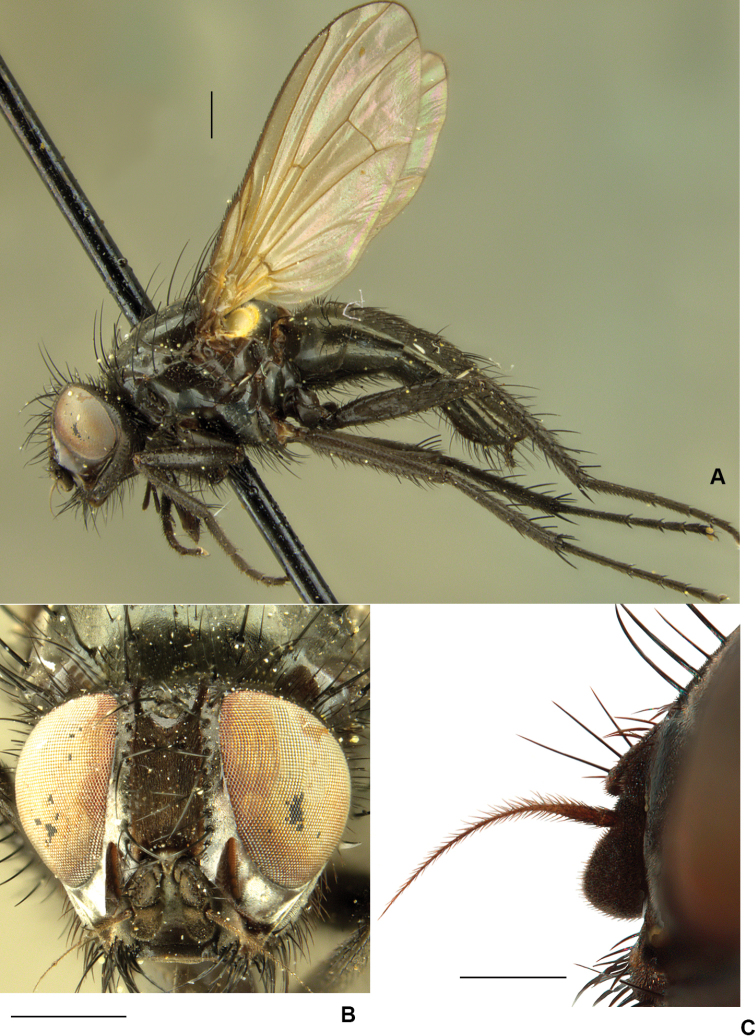
*Drymeia hucketti* sp. nov. **A** female habitus, paratype **B** female head, frontal view **C** female arista. Scale bars: 1 mm (**A, B**); 0.25 mm (**C**).

#### Drymeia profrontalis

Taxon classificationAnimaliaDipteraMuscidae

(Huckett, 1966)

28A23161-C02C-51D8-847C-27ED70F039EF

Figs 6D , 10D , 11C

Pogonomyia profrontalis Huckett, 1966: 293.

##### Type material examined.

*Pogonomyia profrontalis* – ***Holotype*** male labelled “Big Spring/ Shasta Country/ Calif. v-23:41”; “E. G. Linsley/ Collector”; progonomyia/ profrontalis/ n. sp./ holotype [red]”; “California Academy/ of Sciences/ Type No. 10150” (CAS). ***Allotype*** female, same as holotype except “Progonomyia/ profrontalis/ n. sp./ Allotype [orange]; “U.C. Berkeley/ EMEC 69,279 (EMEC). Paratypes: all with “Pogonomyia/ profrontalis/ n. sp./ paratype [blue]”. 4 males, 4 females “Baxter Cal./ Placer Co./ v.20 1952”; “A. T. McClay/ Collector” (UCDC). 5 females “Hope Valley/ Alpine Co Cal/ vi.7 1952”; “R.M. Bohart/ Collector” (UCDC). 1 male, 1 female “10 mi. 3/Johnville/ Cal. Plumas/ Co. vi-12-1961 (UCDC). 2 females “Wright’s Lake/ Eldorado Co./ Calif. VII-2-48”; “R.C. Bynum Collector” (EMEC, UCDC). 1 female “Dutch Flat Cal/ Placer Co./ v-13 1956”; “H.R. Moffitt/ Collector” (UCDC). 1 male “Truckee Cal/ vi-10 1953”; “A.D. Teiford/ Collector” (UCDC).

##### Other material examined.

5 males, 3 females: Nearctic: **USA**: California: Buck’s Lake, Wright’s Lake (EMEC).

##### Distribution.

Nearctic: USA (California).

##### DNA Barcode.

None available.

**Figure 19.**
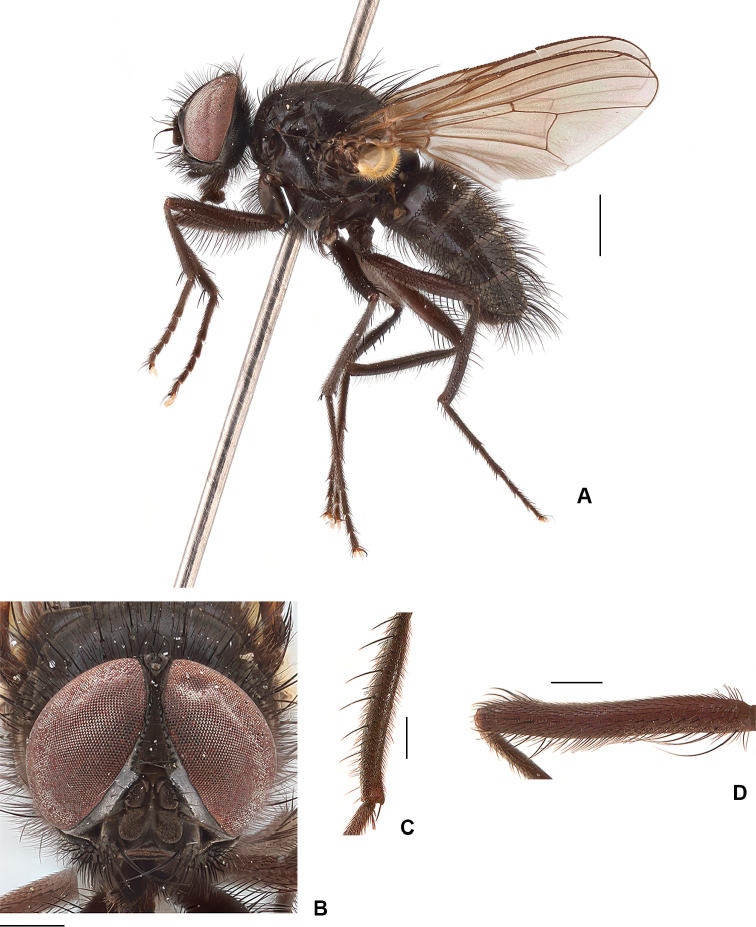
*Drymeia vockerothi* sp. nov. **A** holotype habitus **B** male head, frontal view **C** male hind tibia **D** male mid femur, anterior view. Scale bars: 1 mm (**A, B**) 0.5 mm (**C, D**).

#### Drymeia quadrisetosa

Taxon classificationAnimaliaDipteraMuscidae

(Malloch, 1921)

565CCE66-F245-5A15-9652-F04125856C90

Figs 2G , 5D , 6F

Pogonomyia quadrisetosa Malloch 1919: 77.
Pogonomyia amurensis Lavčiev, 1971: 220

##### Type material examined.

None.

##### Other material examined.

More than 300 males and females from: Nearctic: **Canada**: Manitoba: Churchill, Fort Churchill; Labrador: Cutthroat Harb., Hebron, Nutak; Northwest Territories: Muskox L., Salmita Mines, Tuktoyaktuk, 21 mi E. Tuktoyaktuk; Wholdaia Lake; Victoria Island; Nunavut: Baker Lake, Bathurst Inlet, Cambridge Bay, Chesterfield, Kugluktuk [formerly Coppermine], Landing Lake (7.5 km NW of Rankin Inlet), Padlei; Quebec: Kangirsuk [formerly Payne Bay], Sugluk; Yukon Territory: British Mts, 17 km WNW Burnash Flats, km 159 Dempster Highway, Firth River, Herschel Island; **USA**: Alaska: Cape Thompson, Isabel Pass, Schrader L., Umiat, Unalakleet [Noluk 68N, 160W]. Palaearctic: **Russia**: Taymyr Peninsula: 90 km NW Khatanga; Yamalo-Nenez AO: 73 km NE Labytnangy; Chukotka AO: Pevek; Republic of Buryatia: Baisa; Sakha Republic: 100 km NW Oymyakon (BUIC, CNC, LEM, SZMN).

##### Distribution.

Nearctic: Canada (Manitoba, Northwest Territories, Nunavut, Quebec, Yukon Territory; USA (Alaska). Palaearctic: Russia (Siberia).

##### DNA Barcode.

BOLDBIN: BOLD:AAC1021 (BIN merge with *D. glacialis*). See Suppl. material 1: Table S1 for GenBank accession numbers.

##### Remarks.

See comments about DNA barcodes under *D. glacialis*.

#### Drymeia santamonicae

Taxon classificationAnimaliaDipteraMuscidae

(Huckett, 1966)

7ACF06E4-268D-522E-9EA6-18959D4235DF

Figs 4F , 6G

Pogonomyia santamonicae Huckett, 1966: 294.

##### Type material examined.

*Pogonomyia santamonicae* – ***Holotype*** male labelled “StaMonica Mts./ L.A. Co. Cal./ VII-3-50”; “Pogonomyia/ santamonicae/ n. sp./ holotype [red]”; “California Academy/ of Sciences/ Type No. 10151” (CAS). Allotype female, same as holotype except “Pogonomyia/ santamonicae/ n. sp./ ***Allotype*** [orange]; “U.C. Berkeley/ EMEC 69,280 (EMEC). ***Paratype*** male labelled “Keen Camp Cal/ Riverside Co/ v-18 1951”; “EI Schlinger/ Collector”; “Pogonomyia/ santamonicae/ n. sp./ paratype [blue]” (EMEC).

##### Other material examined.

7 males, 1 female: Nearctic: USA: California: Cleaveland Nat. Forest, Cuyamaca L., Laguna Jct. (EMEC, CNC).

##### Distribution.

Nearctic: USA (California).

##### DNA Barcode.

None available.

#### Drymeia segnis

Taxon classificationAnimaliaDipteraMuscidae

(Holmgren, 1883)

CA54EE0C-A7B1-51DD-B9DF-B932F2961178

Fig. 3B

Aricia segnis Holmgren, 1883: 169.
Pogonomyioides atrata Malloch, 1919: 67.

##### Type material examined.

*Pogonomyioides atrata* Malloch. ***Holotype*** female (with puparium) labelled “Bernard/ Harbour/ N.W.T./July 7. [vertical]”; “Canadian/ Arctic/ Expedition/ F.J. 1915”; “1215”/ “Type/ No. 1180 [red]”; “SLIDE Coll./ A 162 [blue]” [head mounted on slide] (CNC).

##### Other material examined.

More than 500 males and females: Nearctic: **Canada**: Alberta: Eisenhower Jct., Snow Creek Pass (Banff N. P.); Northwest Territories: Holman, Banks Island (Aulavik, Masik river), Sachs Harbour, Tuktoyaktuk; Victoria island; Nunavut: Alex Fiord, Arviat [formerly Eskimo point], Axel Heiberg Island, Baker Lake, Cambridge Bay, Chesterfield, Clyde, Coral Harbour, Devon Island, Ellesmere island, Eureka, Hazen Camp, Kugluktuk, Landing Lake (7.5 km NW of Rankin inlet), Meliadine river, Naujaat [formerly Repulse Bay], Taloyoak [formerly Spence Bay], Tranquary Fjord; Quebec: Inukjuak [formerly Port Harrison]; Yukon Territory: British Mts, Firth River, Herschel Island, Richardson Mts. **USA**: Alaska: Upper Colville River; Colorado: Mt. Evans. **Greenland**: Nedre Midsommer Sö. Palaearctic: **Russia**: Taymyr Peninsula: 90 km NW Khatanga, Ary-Mas cordon, Dixon; Chukotka AO: Wrangel Island; Sakha Republic: 19 km SE Kyusyur, (BUIC, CNC, LEM, SZMN).

##### Distribution.

Nearctic: Canada (Alberta, Manitoba, Northwest Territories, Nunavut, Quebec, Yukon Territory), USA (Alaska, Colorado), Greenland. Palaearctic: Russia.

##### DNA Barcode.

BOLDBIN: BOLD:AAD7664 (BIN merge with several other species, see Fig. 25). See Suppl. material 1: Table S1 for GenBank accession numbers.

##### Remark.

DNA barcodes for *D. segnis* material from Canada and Greenland were very similar to several of the seven species found in the BOLD:AAD7664BIN merge (Fig. 25) and in some cases, identical to those of *D. setibasis*. However, both sexes of *D. segnis* can easily be distinguished from all other species in this BIN by the presence of a haired anepimeron. We would therefore not recommend using COI to discriminate specimens of *D. segnis* from those of other species in this BIN.

#### Drymeia setibasis

Taxon classificationAnimaliaDipteraMuscidae

(Huckett, 1965)

F44F4F0D-476B-5C0B-ACD2-9725BE7147A4

Figs 2D , 6B , 8C

Eupogonomyia setibasis Huckett, 1965: 301.
Thrichopticoides gymnophthalma sibirica Lavčiev, 1971: 220.

##### Type material examined.

*Eupogonomyia setibasis* – ***Holotype*** male labelled “Lady Melville L./ 93°15'W, 69°25'N/ NWT 3.VII 1951”; “Type [red]”; “HOLOTYPE/ CNCNo. 8368 [red]”; “*Eupogonomyia*/ *setibasis* Huck./ det. H.C. Huckett” (CNC). ***Allotype*** female labelled “Spence Bay NWT/ 6. VII. 1951/ A.E.R. Downe”; “Allo [red]”; “ALLOTYPE/ CNCNo. 8368 [red]”; “*Eupogonomyia*/ *setibasis* Huck./ det. H.C. Huckett” (CNC). ***Paratypes***: last 2 labels identical for all and as follows “*Eupogonomyia*/ *setibasis* Huck./ det. H.C. Huckett”; “PARATYPE/No, 8368 (CNC). Remaining data as follows: 2 males same as allotype, 1 male and 1 female same as allotype but 1.VII, 2 males same as allotype but 6.VII, 1 male same as allotype but 22.VII., 1 male and 2 females same as allotype but collected by J.G. Chillcott, 1 female same as allotype but 14.VII., J.G. Chillcott. 1 male “Aklavik, NWT/20.VI-1953/ JS Waterhouse”; 1 female “Clyde, Baffin Is./ N.W.T. 27-VI-1958/ G.E. Shewell”. 1 female “Herschel Is. Y. T./ 11-VII-1953/ J.S. Waterhouse”. 1 female “Holman, N.W.T./ Victoria Is./ 26 VI 1952/ D.P. Gray”. 1 male and 5 females “Padley N.W.T./ 24-VII-1950/ R.E. Duckworth”. 1 male same as previous but 27-VII-1950. 1 female “Firth River, Y.T./14-VII-1956” 2 females, same as previous but 17-VII-1956 and 2-VIII-1956. 1 female “Baker Lake N.W.T./ 13.VIII 1947/ T.N. Freeman”. 1 female “Eskimo Point/ N.W.T. VIII 1950 [2 handwritten above 0]/ G.R. Roberts”.

##### Other material examined.

More than 50 males and females from Nearctic: **Canada**: Northwest Territories: Tuktoyaktuk; Nunavut: Arviat [formerly Eskimo point], Cambridge Bay, Landing Lake (7.5 km NW of Rankin inlet), Taloyoak [formerly Spence Bay]; Yukon Territory: 17 km WNW Burwash Flats. Palaearctic: **Russia**: Taymyr Peninsula: Ary-Mas cordon, 12.5 km S. Dikson settlement, 90 km NW Khatanga; Altai Republic; Chukotka AO: Wrangel Island (BUIC, CNC, LEM, SZMN).

##### Distribution.

Nearctic: Canada (Northwest Territories, Nunavut, Yukon Territory), USA (Alaska). Palaearctic: Armenia, Georgia, Kyrgyzstan, Russia.

##### DNA Barcode.

BOLDBIN: BOLD:AAD7664 (BIN merge with several other species, see Fig. 25). See Suppl. material 1: Table S1 for GenBank accession numbers.

##### Remarks.

See comments under *D. segnis* and *D. cantabrigensis*.

#### Drymeia similis

Taxon classificationAnimaliaDipteraMuscidae

(Malloch, 1918)

4D93934E-4E3A-5106-884C-18D5EF496EAD

Figs 2E , 6I , 10C , 11A

Pogonomyia similis Malloch, 1918: 279.

##### Type material examined.

*Pogonomyia similis* Malloch – ***Holotype*** male labelled “Top of Las/ Vegas Range/VI.28.02 NM”; “HoloTYPE/6200 [red]”; “HoloTYPE/ Pogonomyia/ MINOR/ Mall. [red]” (ANSP). ***Paratype*** male labelled “Elev. 4800”; “*Pogonomyia*/ PARATYPE/♂ *similis* Mal./ No. 2748 [yellow]”; Bozeman Mont./ July 7- 1902”; “PARATYPE/ *Pogonomyia*/ *similis*/♂ Malloch [blue]” (CNC).

##### Other material examined.

Nearctic: **Canada**: Alberta: Acme, Banff, 20 mi W Calgary, Drumheller, Eisenhower Jct., Elkwater Lake, Frank, Hinton, Lethbridge, 15 km east Morley, Waterton, British Columbia: Atlin, Cathedral Mtn, Clinton, Crowsnest, Liard Hot Spg., Vernon, Victoria; Manitoba: Carberry, Gimli, Husavick, Pierson, Reynolds, 30 mi N Roblin, Taulon[a], Virden; Northwest Territories: Fort Liard, Hay River; Ontario: Moosonee, Thor Lake; Quebec: Mistassini, Rupert House; Saskatchewan: Big River, Cypress Hills, Kenosee, Melfort, Prince Albert; Yukon Territory: Dawson. **USA**: Colorado: Chicago Cr., Estes Park, Gilpin, Idaho Springs, Jefferson, Loveland Pass, Mt. Evans (Doolitle Ranch, Echo Lake), Nederland, Niwot Ridge; Utah: Daniels Pass; Wyoming: Togwotee Pass, Union Pass Road. **Greenland**: Nedre Midsommer Sö. (CNC).

##### Distribution.

Nearctic: Canada (Yukon Territory and British Columbia to Newfoundland and Labrador), USA (Alaska to New Mexico), Greenland.

##### DNA Barcode.

BOLDBIN: BOLD:AAG1776. See Suppl. material 1: Table S1 for GenBank accession numbers.

##### Remark.

All three specimens of *D. similis* in our DNA barcoding data set were from Saskatchewan (Canada) with p-distances ranging from 0.0% to 0.16% (Fig. 25).

#### Drymeia spinitarsis

Taxon classificationAnimaliaDipteraMuscidae

(Aldrich, 1918)

5CD8485A-BACD-5737-A18F-805F5EFCCBBC

Figs 4D , 5B, C , 8A

Pogonomyia spinitarsis Aldrich, 1918: 184.
Drymeia longiseta Sorokina & Pont, 2015: 181. syn. nov.

##### Type material examined.

*Drymeia longiseta* Sorokina & Pont. ***Holotype*** male labelled “Russia, Republic Altai/ Kosh-Agash area, 7 km NW/ Kuray, 2251 m, Kurayskiy/ mt. ridge, 50°17'N, 87°51'E/ Coll. V. Sorokina, 17.07.2013”; “Holotype/ Drymeia longiseta ♂/ Sorokina & Pont sp.n. [red]” (SZMN). ***Paratypes***: 1 male, same as previous but “Paratype/ Drymeia longiseta ♂/ Sorokina & Pont sp.n. [red]” (BUIC). 1 male, 2 females labelled “Russia, Republic Altai/ Kosh-Agash area S slope/ of Kurayskiy mt. ridge,/ 2726 m. 50°18'N, 87°51'E/ Coll. V. Sorokina, 19.07.2013”; “Paratype/ Drymeia longiseta ♂[or ♀]/ Sorokina & Pont sp. n. [red]” (BUIC).

##### Other material examined.

Over 400 males and females from: Nearctic: **USA**: Colorado: Corona Pass, Cottonwood Pass, Estes Park, Loveland Pass, Mt. Evans, Nederland, Niwot Ridge, Summit Lake (Mt. Evans) (CNC).

##### Distribution.

Nearctic: USA (Colorado). Palaearctic: Russia (Altai-Sayan region).

##### DNA Barcode.

BOLDBIN: BOLD:ACT4320. See Suppl. material 1: Table S1 for GenBank accession numbers.

##### Remarks.

The comparison of type material from *D. longiseta* Sorokina and Pont from Russia with a series of high elevation specimens from Colorado, USA, housed in the CNC and matching the original description of *D. spinitarsis* has led us to recognise *D. longiseta* as a junior synonym of *D. spinitarsis*. This change effectively expands the distribution of *D. spinitarsis* to the Palaearctic region and while DNA barcodes are currently only available for Russian specimens, *D. spinitarsis* is such a large, distinctive species (strong spines on mid tarsomere 1, distinctive chaetotaxy of fore and mid coxae in the male, high elevation distribution) that we are quite confident in this new synonymy.

#### Drymeia vockerothi sp. nov.

Taxon classificationAnimaliaDipteraMuscidae

A7AB4D14-736D-5B9C-9E94-7EF7C16B5C45

http://zoobank.org/294CCCE6-6029-4877-AA3E-19E8BFDD1C0A

**Figure 20.**
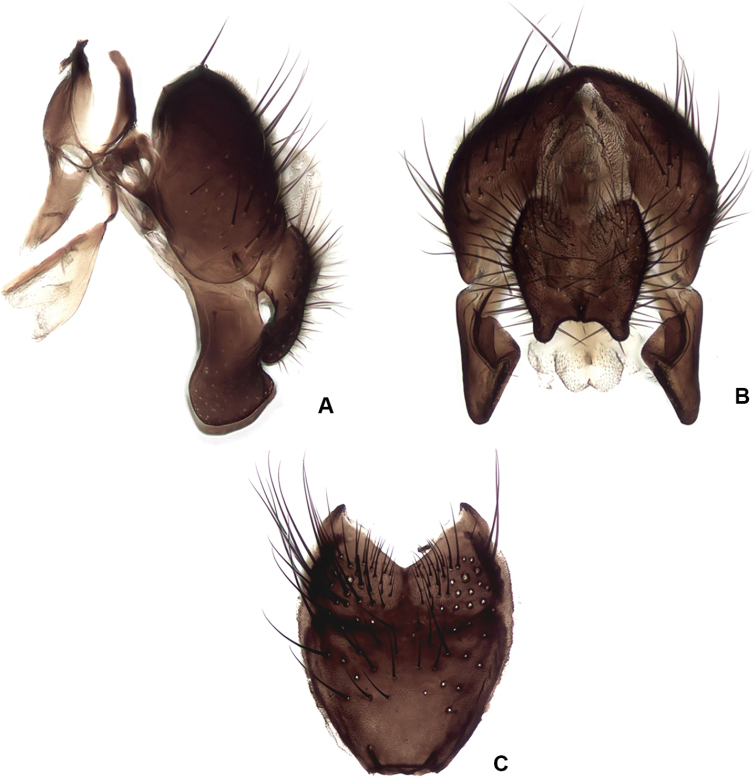
*Drymeia vockerothi* sp. nov. **A** male terminalia, lateral view **B** posterior view **C** sternite V.

**Figure 21.**
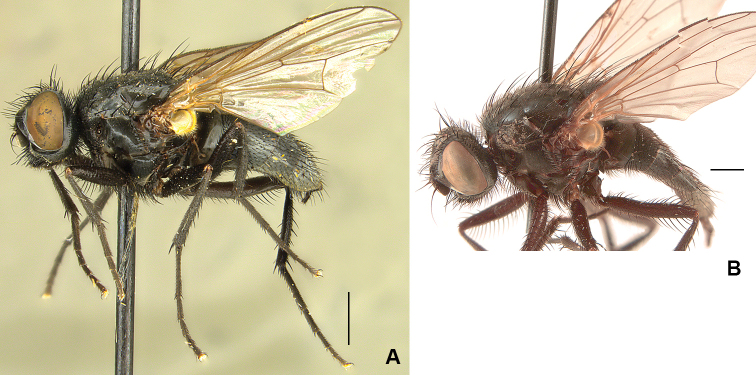
*Drymeia vockerothi* sp. nov. **A** female habitus, paratype **B** female, dorso-lateral view. Scale bars: 1 mm.

Figs 1D , 2F , 4C , 6C , 19 , 20 , 21

##### Type material.

***Holotype*** male labelled “Rigaud, QUE./ 11.VI. 1981/ J.R. Vockeroth”; “Summit of/ Mt. Rigaud”; “CNC 91243”; “HOLOTYPE/ Drymeia vockerothi ♂/ Savage & Sorokina [red]” (CNC). ***Paratypes***: all with “PARATYPE/ Drymeia vockerothi/ Savage & Sorokina [yellow]” (CNC unless otherwise indicated): 4 males same as holotype. 1 male same as holotype except (BUIC). 1 pair in copula and 1 male labelled “QUE Cté Vaudreuil [currently MRC Vaudreuil-Soulange]/ summit Mt Rigaud/ 6.VIII.1992/ D.M. Wood 220m” (SZMN). 1 male labelled “QUEBEC, Summit/ Rigaud Mtn./ 2.VI.1981/ D.M. Wood”. 1 male labelled “QUE., Rigaud/ Summit Mtn./ 18.VI.1986/ H.C. Walther”. 3 males labelled “Masham Twp. [currently La Pêche], QUE./ Gatineau Co./ 10–20 VII.1974/ D.M. Wood”. 1 male same as previous except 28–31.VII.1974. 2 males same as previous except 27–31.V.1974. 1 male same as previous except 22–26.VI.1974. 1 male same as previous except 20–24.V.1974. 1 male same as previous except 25–31.VII.1974. 1 male same as previous except 1–5.VII.1974. 1 male same as previous except 6–8.VII.1974.1 female labelled “Summit King Mt./ Old Chelsea QUE/ 1150' 24.VIII.67/ J.R. Vockeroth” (BUIC). 1 male labelled “SH58; June 27/65/ St Hilaire/ P.Q. Canada” [collector unknown]. 1 female labelled “Duncan Lake,/ Nr Rupert, Que/ 31.VII.1971/ J.F. McAlpine”. 1 female labelled “Kouchibouguac N. P./ N. B. 12.VII.1978/ S.J. Miller/ Code-7267M”. 1 female labelled “2.Mi.N./ Metcalfe, Ont./28.VI.1982/B.E. Cooper”. 1 male labelled “Metcalfe, ONT./ 2.VIII.1984/ B.E. Cooper”. 1 male, same as previous except 24.VII.1984.1 female labelled “Maynooth ONT./10 VII.1965/ J.F. McAlpine”.

##### Etymology.

The species name is a patronym in honour of famous dipterologist John R. Vokeroth (Canada), who collected the holotype and several paratypes and designated these specimens as a “new species near *D. neoborealis*”.

##### Diagnosis.

Large glossy black species with short proboscis, very narrow parafacial in lateral view, arista almost bare, prealar weak or absent, 2+3 or 4 *dc* and well developed presutural *acr.* Males with narrow frons (Fig. 19B), F2 with at most a few long *av* near base and with ventral apical process of T3 short but distinct (Fig. 19C). This species is similar to *Drymeia neoborealis* (Snyder, 1949) but can be distinguished from it in both sexes by the presence of strong presutural *acr* and a narrow parafacial in lateral view.

**Male.** Body length: 7.2–9.5 mm; wing length: 6.0–6.7 mm (Fig. 19A).

***Head*:** Ground colour black; eye bare; fronto-orbital plate and parafacial yellowish silver pruinose; face grey, gena and lower occiput grey pruinose; frons at narrowest point 1.5–2.5 × width of anterior ocellus with fronto-orbital plates touching or nearly touching (Fig. 19B); parafacial very narrow in lateral view, < 1/2 × width of first flagellomere along full length; lower margin of the face equal to or slightly behind lower level of profrons in lateral view; gena at narrowest point 0.5 × length of first flagellomere, densely setulose and with a group of upcurved setae on anterior part of genal dilation; 14–16 frontal setae (including interstitials) reaching almost to anterior ocellus; antenna black; first flagellomere 1.3 × as long as wide; arista swollen near base and almost bare, with longest hair much shorter than basal diameter of arista; palpus black; proboscis broad with prementum shorter than palpus, mostly undusted and glossy; labella large and fleshy.

***Thorax*:** Ground colour black; scutum, postpronotum, notopleuron, postalar callus, pleuron and scutellum black and shiny, mostly undusted; large undusted glossy patches on anterior part of katepisternum and ventral area of meron; anepimeron and katepimeron bare; notopleuron densely setulose; *acr* 2 or 3+4 or 5, stronger than ground setulae and with preapical series at least as long as preapical *dc*; 2+4 *dc*; prealar weak or absent, always much shorter than 2^nd^ notopleural.

***Legs*:** Black; T1 with 1 or 2 *pv*; F2 straight, slightly flattened anterodorsally, with 3 or 4 long delicate upcurved *av* on basal 1/3 and short setae on remaining *av* surface (Fig. 19D), 2 or 3 preapical *pd-p*, and a row of fine dense *pv*; T2 without *av*, 5–7 *pd* and 2 or 3 *pv*; F3 with *av* row stronger and longer than width of femur on apical 1/2, without *pv*; T3 clothed on most surfaces with short erect setae, 4–6 *pd* of irregular length on basal 1/2 and occasionally 2 or 3 short *pd* on apical 1/2, ventral apical process short but distinct, apical pv absent (Fig. 19C).

***Wing*:** Light brownish, yellow at base; basicosta and tegula black; costal spinules weak and costal spine reduced; calypters with membrane and edges yellow.

***Abdomen*:** Conical, ground colour black; densely grey dusted with narrow black median vittae on tergites II–V; sternite I bare; sternite V as in Fig. 20C.

***Terminalia*:** Fig. 20A, B.

**Female.** Body length: 7.5–9.0 mm; wing length: 6.2–6.8 mm (Fig. 21A).

Differs from the male as follows:

***Head*:** Ground colour black with grey dusting; frontal vitta brown with grey dusting; frontal triangle indistinct, ocellar triangle undusted and shiny; parafacial entirely dusted, without shiny patch near base of antenna; frons at midpoint 0.36 × as wide as head and approximately 0.85 × as long as wide; fronto-orbital plate wide, at midpoint 0.4 × as wide as frontal vitta; seven medioclinate frontal setae (including several interstitials), three orbital setae, the upper two reclinate and lateroclinate, the lower one proclinate.

***Thorax*:** Ground colour as in male but with light grey dusting (Fig. 21B); *acr* as in male but often in irregular row.

***Legs*** (chaetotaxy described in full): T1 with 1 or 2 *pv*; F2 not flattened anteroventrally, with 2 or 3 short *av*, 2 or 3 short *v* on basal 1/3, and with a few short fine *pv* on apical 1/3; T2 with 1 or 2 *ad*, 3–5 *pd* and 1 or 2 *pv*; F3 with *av* row stronger on apical 1/2, without *pv*; T3 with 3 or 4 short *av*, 3 or 4 *ad*, and 3–6 *pd*, apical *pv* absent.

***Wing*:** As in male.

***Abdomen*:** Without distinct median vittae.

##### Distribution.

Nearctic: Canada (New Brunswick, Ontario, Quebec).

##### DNA Barcode.

None available.

#### Drymeia woodorum sp. nov.

Taxon classificationAnimaliaDipteraMuscidae

4D4F2F1E-4BD7-5AED-ABEA-DE3A38F416CF

http://zoobank.org/73505025-739A-476C-9B2C-AF548257085B

**Figure 22.**
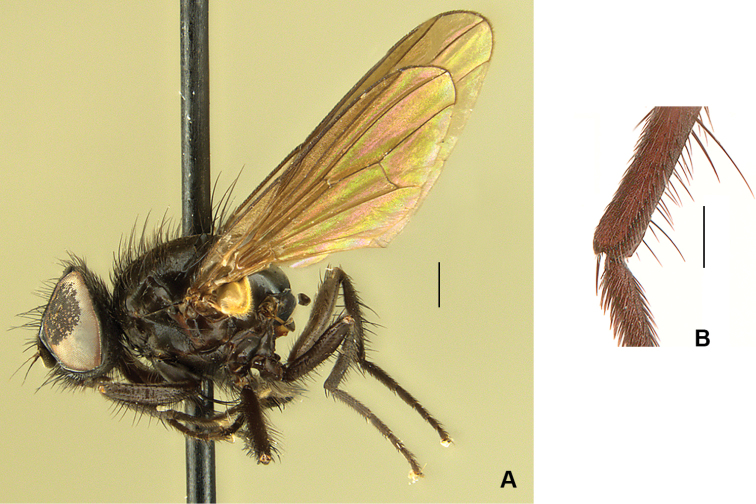
*Drymeia woodorum* sp. nov. **A** holotype habitus **B** male hind tibia. Scale bars: 1 mm (**A**); 0.25 mm (**B**).

**Figure 23.**
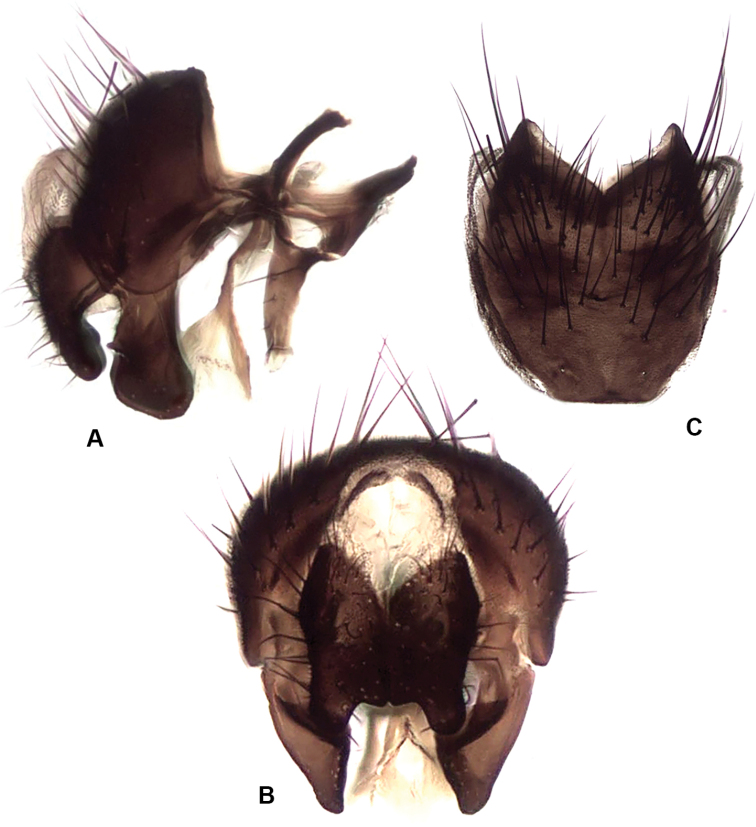
*Drymeia woodorum* sp. nov.: male terminalia **A** lateral view **B** posterior view **C** sternite V.

**Figure 24.**
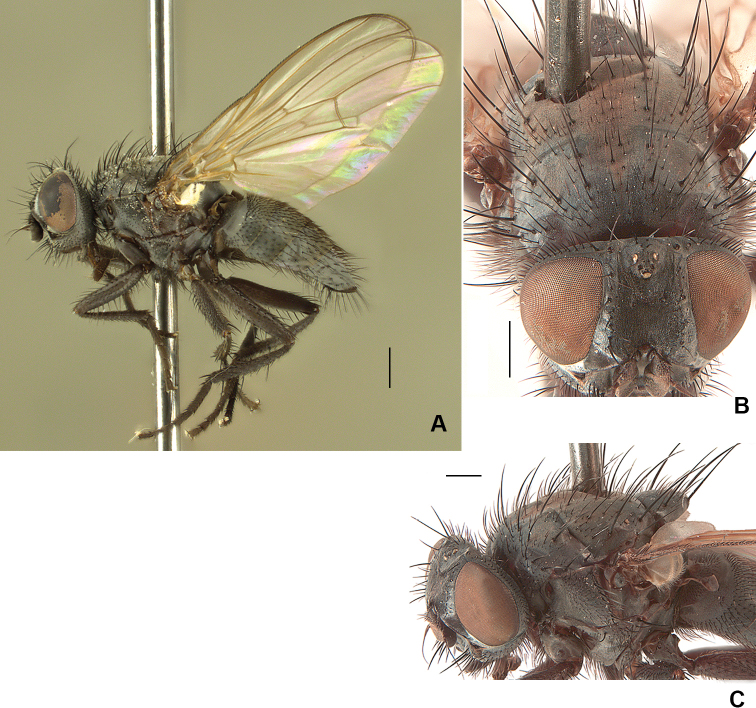
*Drymeia woodorum* sp. nov. **A** female habitus, paratype **B** female frons and scutum, dorsal view **C** female, dorso-lateral view. Scale bars: 1 mm (**A**); 0.5 mm (**B, C**).

Figs 2J , 22 , 23 , 24

##### Type material.

***Holotype*** male labelled “MI. 87, Y.T./ Depmster Hwy./ 4–8.VIII.1973/ G. & D.M Wood”; “HOLOTYPE/ Drymeia woodorum ♂/ Savage & Sorokina [red]” (CNC). ***Paratypes***: all with “PARATYPE/ Drymeia woodorum/ Savage & Sorokina [yellow]” (CNC unless otherwise indicated): 1 female labelled “Swim Lakes, Y.T./ 133°, 62°13'/ 3200' 25.VI.60/ J.E.H. Martin”. 1 female, same as previous except 12.VII.60 and (BUIC). 1 female labelled “La Force L., Y.T./ 132°20', 62°30'/ 3300' 10.VII.60/ J.E.H. Martin”. 1 female labelled “La Force L., Y.T./ 132°20', 62°41'/ 3300' 29.VI.60/ J.E.H. Martin”. 1 female, same as previous except 5.VII.60. 1 female, same as previous except 10.VII.60. 2 females, same as previous except 13.VII.60. 2 females labelled “La Force L., Y.T./ 132°20', 62°41'/ 3300' 25.VI.60/ E.W. Rockburne”. 1 female, same as previous except 26.VI.60. 1 female, same as previous except 11.VII.60. 1 female, same as previous except 12.VII.60. 1 female, same as previous except 12.VII.60 and (BUIC). 1 female labelled “Whitehorse, Y.T./ 29/VIII 1949/ L.C. Curtis”.

##### Etymology.

The species name is a patronym in honour of the late Canadian dipterologist D. Monty Wood and his wife Grace Wood (Canada), who collected the holotype.

##### Diagnosis.

Small grey species with short proboscis, narrow frons and narrow parafacial in lateral view (Fig. 22A), arista almost bare, prealar absent in male and weak in female, 2+4 *dc* and well developed presutural *acr.* Male with patch of four or five very long, strong apical bristles on fore coxa and with a row of *ad* on apical 2/3 of T2. This species is similar to *Drymeia neoborealis* and *Drymeia vockerothi* sp. nov. but can be distinguished from them in both sexes by the smaller size, in the male by the bristles of the fore coxa and T2, and in the female by the combination of strong presutual *acr*, completely dusted prementum, and parafacial in lateral view nearly as wide as width of first flagellomere.

##### Description.

**Male.** Body length: 5.5 mm wing length: 4.5 mm.

***Head*:** Ground colour black; eye bare; ocellar triangle, fronto-orbital plate and parafacial silvery pruinose; face grey, gena and lower occiput light grey pruinose; fronto-orbital plates touching in the middle; frons at narrowest point as wide as width of anterior ocellus; parafacial very narrow in lateral view, < 1/2 width of first flagellomere along full length (Fig. 22A); lower margin of the face equal to or slightly behind lower level of profrons in lateral view; gena at narrowest point 0.5 × length of first flagellomere, densely setulose and with a group of upcurved setae on anterior part of genal dilation; 12 frontal setae (including interstitials) reaching almost to anterior ocellus; antenna black; first flagellomere 1.3 × as long as wide; arista swollen near base and almost bare, with longest hair much shorter than basal diameter of arista; palpus black; proboscis short, with prementum shorter than palpus and heavily dusted; labella large and fleshy.

***Thorax*:** Ground colour black; scutum without distinct vittae; postpronotum, notopleuron and postalar callus with dense brown dusting; pleuron brownish grey dusted; undusted glossy patches on most anterior part of katepisternum and on a small ventral area of meron; anepimeron and katepimeron bare; notopleuron densely setulose; *acr* 2 or 3 + 4 or 5 (+1 prescutellar), stronger and longer than ground setulae (and with preapical series at least as long as preapical *dc*); 2 or 3+4 *dc*; prealar absent.

***Legs*:** Black; fore coxa with patch of four or five very long, strong apical bristles, at least as long as length of coxa; T1 with 1 or 2 *pv*; F2 with a row of short *av* on apical 1/3, 5 or 6 *a* on basal 1/2, these longer than width of femur, 3 preapical *pd-p*, and dense fine *pv* over most of the surface; T2 without *av*, with a row of *ad* on apical 2/3, these longer near apex, 4 *pd* and 2 *pv*; F3 with *av* row stronger on apical 1/3, without *pv* except for a few hairs near base; T3 with 3 *av*, 3 long *ad*, a row each of short erect uneven *a* and *ad*, 2 long and 2 or 3 short *pd*, ventral apical process very short but distinct, apical *pv* absent (Fig. 22B).

***Wing*:** Mostly dark brown; basicosta and tegula black; costa with short weak spinules and costal spine reduced; calypters with membrane and edges deep yellow.

***Abdomen*:** Conical, ground colour black; densely grey dusted with a black median vitta on tergites II–V; sternite I bare; sternite V as in Fig. 23B.

***Terminalia*:** Fig. 23A, B.

**Female.** Body length: 4.9–5.5 mm wing length: 4.3–4.7 mm (Fig. 24A). Differs from the male as follows:

***Head*:** Ground colour grey with grey dusting; frontal vitta black with brownish dust; frontal triangle indistinct, ocellar triangle covered with heavy grey or brown dust; parafacial completely dusted but with large area near base of antenna appearing velvety brown in anterior and lateral views (Fig. 24B, C); frons at midpoint approximately 0.4 × as wide as head and approximately 0.75 × as long as wide; fronto-orbital plate narrow, at midpoint 0.16–0.2 × as wide as frontal vitta; gena approximately as high as length of first flagellomere; five or six medioclinate frontal setae (including interstitials), three orbital setae, the upper two reclinate and lateroclinate, the lower one proclinate.

***Thorax*:** Scutum with whitish grey dust and, when viewed from the front, with 3 brown vittae with diffuse margins (Fig. 24B); postpronotum, notopleuron, postalar lobe and pleuron grey dusted; *acr* as in male but with slightly uneven rows; prealar distinct but weak in all females from the type series, always much shorter than 2^nd^ notopleural.

***Legs*** (chaetotaxy described in full): Fore coxa without patch of long strong bristles; T1 with 1 or 2 *pv*; F2 with a row of short *av*, no longer than width of femur and a row of delicate *pv-v*, no longer than diameter of femur; T2 usually with a row of 5–7 irregular *pd*, (these sometimes reduced) and 1 *pv*; F3 with *av* row stronger on apical 1/3, without *pv* except for a few hairs near base, T3 with 2–3 *av*, 5–6 short irregular *ad* and 5–6 *pd*, apical *pv* usually absent but if visible, then no longer than 1/2 the length of apical *av*.

***Wing*:** Clear with slight pale brown to pale yellow tinge, veins pale brown; calypters whitish.

***Abdomen*:** Densely whitish grey dusted with slight brownish tinge along posterior margins of tergites II–IV.

##### Distribution.

Nearctic: Canada (Yukon Territory).

##### DNA Barcode.

None available.

##### Remarks.

See notes on problematic taxa.

## Notes on problematic North American taxa

Huckett (1965a: 297) provided a key (but no descriptions) to females for three unnamed species of northern *Drymeia* with a short prealar, short proboscis and proclinate anterior orbital setae (as *Trichopticoides* sp. A, sp. B, sp. C). While we could not trace the specimens listed by the author, females of *Drymeia woodorum* sp. nov. (Fig. 24A–C) would key out to *Trichopticoides* species B (recorded from Alaska and the Yukon Territory) and one paratype from Whitehorse, Yukon Territory, bears a yellow label written “Trichopticoides’’ in what appears to be Huckett’s handwriting. The taxa referred to as *Drymeia* sp.1 (BOLD:ACA9236) and *D.* sp. 2 (BOLD:ACZ1539) in the present work (Fig. 25) would also have keyed out to “*Trichopticoides*” in Huckett (1965a); specimens of *D.* sp. 1 were too damaged for additional assessment but those of *D.* sp. 2 (two females from Kluane, northern British Columbia) would key out to “*Trichopticoides*” sp. C, recorded from nearby Alaska. Unfortunately, as we have not located the specimens listed in Huckett (1965a) we remain unable to assess if one or more of his unnamed species are a true match for some of our material.

**Figure 25.**
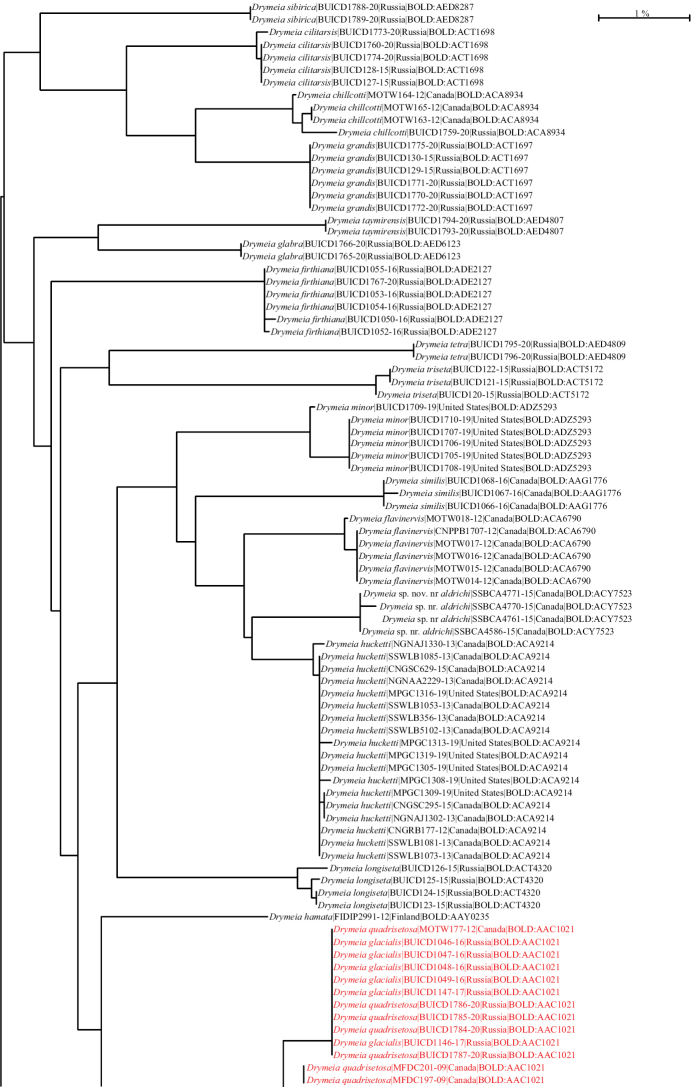
Neighbour-joining tree based on p-distance of all COI sequences (> 550 bp) for which specimens were examined. Each terminal includes the species name, BOLD process ID number, country of collection, and Barcode Index Number. Red text indicates the presence of more than one named species grouped under a single BIN (BIN merge).

**Figure 25.**
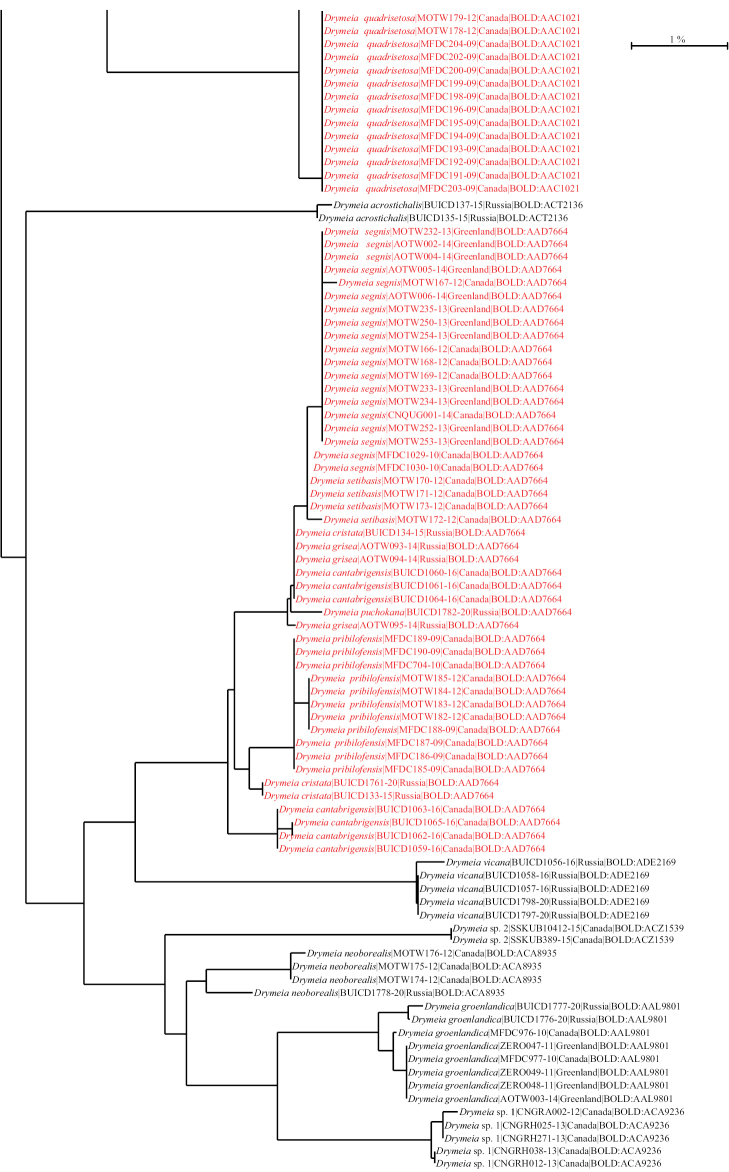
Continued.

*Drymeia vockerothi* sp. nov. is another species with a short prealar, short proboscis and proclinate orbital setae but it is much larger than the other taxa discussed above and does not fit the features listed in the key or the distribution of any *Trichopticoides* spp. mentioned in Huckett (1965a).

### Morphology

Three new synonymies are established here (the recognition of *D. longiseta* Sorokina & Pont, 2015 as a junior synonym of *D. spinitarsis* (Aldrich, 1918), of *D. rivalis* (Huckett, 1966) as a junior synonym of *D. amnicola* (Huckett, 1966), and of *Drymeia alpicola* (Rondani, 1871) as a junior synonym of *Drymeia glacialis* (Rondani, 1866)). These synonymies are based exclusively on morphology and details are provided in the checklist section of this work.

Most species of *Drymeia* show distinctive and discrete morphological differences between species (at least in the males). However, the four species described in Huckett (1966) from California turned out to be especially challenging. Specimens of *D. profrontalis* (Huckett, 1966) can usually be distinguished from other similar species but we found that even following the synonymy of *D. rivalis* with *D. amnicola*, some specimens (mostly females but even some males) could not be unambiguously assigned to either *D. amnicola* or *D. santamonicae* (Huckett, 1966). While the type series for these taxa are generally long, we could not find recently collected material; we therefore plan to prioritise western California in future collecting efforts.

### DNA barcoding

A total of 175 sequences were included in the DNA barcode data set (Suppl. material 1: Table S1). The majority (150) were from specimens assigned to named species while the remaining 25 could not be matched to any known taxa. This material came from Canada, Russia, Greenland, the United States, and Finland; COI sequences were assigned to 24 BINs in BOLD (Fig. 25).

Eighteen named species and four unknown taxa matched a single BIN. These numbers were compiled after we re-examined material (all paratypes) from BOLD:ACT1697 (males and females assigned to *D. grandis* Sorokina & Pont, 2015) and BOLD:ACT1698 (a mixture of males *D. cilitarsis* Sorokina & Pont, 2015, and females originally assigned to *D. grandis*). The female of *D. cilitarsis* is currently undescribed and the re-examination of specimens from these two BINs led us to the conclusion that the paratype series of *D. grandis* is mixed, containing properly identified females as well as some belonging to *D. cilitarsis* (both taxa have similar distributions). The description of the female of *D. cilitarsis*, a Palaearctic species, is outside the scope of the present work and will be completed in a separate contribution.

Following the examination of material from the four BINs containing specimens we could not assign to known species based on morphology we described one new species, *Drymeia hucketti* sp. nov. (BOLD:ACA9214). Due to the very poor condition of available specimens (BOLD:ACA9236), combined with a lack of males for BOLD:ACZ1539 we chose to leave these two clusters unassigned as *Drymeia* sp. 1 and *Drymeia* sp. 2, respectively (Fig. 25), until more material becomes available. Both were relatively small (< 6.5 mm), had short mouthparts, non-projecting faces, prealar absent, and 2+4 *dc*; in males of sp. 1, the apical ventral process on T3 was very short (see notes on problematic North American taxa for additional details).

The four specimens (two from each sex) available for BOLD:ACY7523 (Drymeia sp. nr. aldrichi), were most similar to *D. aldrichi* Malloch, 1918 but differed slightly from material examined (holotype male + two females from same series) in the following features: females with very short *av* and *pv* near the base of the mid femur and with tarsomere 5 not flattened, males with projection of frons slightly more pronounced and with slightly longer *pv* on the apical 1/2 of the mid femur. Since no DNA barcodes were available for material matching the morphology of specimens of *D. aldrichi* examined here, we chose to leave this cluster unresolved as we are unable at present time to determine if the material from BOLD:ACY7523 belongs to a new distinct species or if *D. aldrichi* is more variable morphologically than currently understood.

While the congruence between BIN assignment and morphology was good for 71% (22/31) of species studied here (including the three unnamed taxa), a significant proportion of species (9/31) presented no or very low interspecific distance to other morphologically distinct taxa. These taxa were grouped under two BIN “merges” including two (BOLD:AAC1021) and seven (BOLD:AAD7664) species (Fig. 25). In both cases, obvious and discrete morphological differences (at least in the males) exist between all species from these mixed clusters (see identification keys; remarks under *D. cantabrigensis*, *D. glacialis*, *D. pribilofensis* and *D. segnis*; and Sorokina and Pont (2015)).

Low interspecific distance between sequences from Canadian specimens of *D. pribilofensis* and *D. segnis* had previously been flagged by Renaud et al. (2012) but our wider taxon sampling uncovered a more complex issue, now with seven species clustering together in BOLD:AAD7664 with p-distances ranging from 0.0% to 1.83%. Of these taxa, only *D. cantabrigensis* is restricted to the Nearctic region. *Drymeia pribilofensis*, *D. setibasis*, and *D. segnis* are Holarctic and the remaining species (*D. cristata* Sorokina & Pont, 2015, *D. grisea* Sorokina & Pont, 2015 and *D. puchokana* Sorokina & Pont, 2015) are known only from the eastern Palaearctic region. *Drymeia pribilofensis* was the only species from BOLD:AAD7664 where all sequences in the data set shared unique nucleotides at several positions in the alignment (50, 58, 300, 475) but these differences were not enough to provide an unambiguous species-level match in BOLD and classify these sequences in a separate group under the BIN system (Ratnasingham and Hebert 2013).

Male specimens were included in our dataset for all species involved in the two BIN merges except *D. puchokana*; we therefore exclude misidentification as a possible explanation for the mismatches between BIN assignment and morphology reported here. Low genetic divergence in COI sequences between closely related species has been reported in the literature for different groups of animals (e.g., Whitworth et al. 2007; Gibbs 2018; Tizard et al. 2019) and can result from a number of non-exclusive causes such as polymorphism, hybridisation and/or incomplete lineage sorting (Funk and Omland 2003). Additional data based on other genetic markers and/ or reproductive compatibility experiments will therefore be required before modifying the current taxonomy for members of the two BIN merges reported here in *Drymeia*.

## Conclusions

We found both advantages and limitations to using DNA barcodes and BIN assignments for the identification of *Drymeia* species. They allowed us to resolve the issue of male/female associations in the Palaearctic species *D. grandis* and *D. cilitarsis*, to discover one new species, *D. hucketti* sp. nov., and to focus our attention on three potentially new taxa (*Drymeia* sp. 1, *D.* sp. 2, D. sp. nr. aldrichi). BINs were also a good match for 22 of the 31 species included in our data set, regardless of geographical distance. However, the nine remaining species could not be distinguished from at least one other species; we therefore caution against using BIN assignments based on COI DNA barcoding as the only determination tool for *Drymeia* material without prior knowledge of its limitations for certain species groups. This is especially important for the Nearctic fauna where only 65% (11/17) of the species represented in our DNA barcode library (including the three unnamed taxa) have a match between BIN and morphology while the remaining six show no or very low sequence divergence with one or more morphologically distinct species.

Considering the taxonomic changes presented here, the North American fauna of *Drymeia* now includes 24 species; 22 are found in the Nearctic region, *D. ponti* sp. nov. is recorded only from Neotropical Mexico, and *D. aterrima* has been recorded from both the Nearctic and Neotropical regions. Furthermore, the synonymy of *D. longiseta* with *D. spinitarsis* now brings the number of Holarctic species to ten. Whereas the present study provides the first comprehensive taxonomic treatment of *Drymeia* for North America, ten of the 24 named species of *Drymeia* are still missing DNA barcodes and some additional work remains. The most important gap in knowledge concerns the fauna of the southwestern United States and Mexico and the collection of additional specimens from those areas are likely to result in further changes to the taxonomy of the genus.

## Supplementary Material

XML Treatment for Drymeia

XML Treatment for Drymeia aldrichi

XML Treatment for Drymeia amnicola

XML Treatment for Drymeia aterrima

XML Treatment for Drymeia cantabrigensis

XML Treatment for Drymeia chillcotti

XML Treatment for Drymeia firthiana

XML Treatment for Drymeia flavinervis

XML Treatment for Drymeia glacialis

XML Treatment for Drymeia groenlandica

XML Treatment for Drymeia hucketti

XML Treatment for Drymeia latifrons

XML Treatment for Drymeia minor

XML Treatment for Drymeia neoborealis

XML Treatment for Drymeia ponti

XML Treatment for Drymeia pribilofensis

XML Treatment for Drymeia profrontalis

XML Treatment for Drymeia quadrisetosa

XML Treatment for Drymeia santamonicae

XML Treatment for Drymeia segnis

XML Treatment for Drymeia setibasis

XML Treatment for Drymeia similis

XML Treatment for Drymeia spinitarsis

XML Treatment for Drymeia vockerothi

XML Treatment for Drymeia woodorum

## Supplementary materials

Supplementary material 1 Table S1 Data type DNA barcoding data set Explanation note Details of specimens included in the DNA barcoding data set. https://binary.pensoft.net/file/520368This dataset is made available under the Open Database License (http://opendatacommons.org/licenses/odbl/1.0/). The Open Database License (ODbL) is a license agreement intended to allow users to freely share, modify, and use this Dataset while maintaining this same freedom for others, provided that the original source and author(s) are credited.Jade Savage, Vera S. Sorokina
